# Time-restricted eating as a potential strategy for healthy lifespan: an evaluation of current evidence

**DOI:** 10.3389/fmed.2025.1701888

**Published:** 2026-01-12

**Authors:** Heying Wu, Yuqing Shi, Zixu Wang, Zhenya Wei, Chong Cui, Huazhong Xiong, Zeyu Wang, Jixiang Ren

**Affiliations:** 1 College of Traditional Chinese Medicine, Changchun University of Chinese Medicine, Changchun, China; 2 College of Integrated Chinese and Western Medicine, Changchun University of Chinese Medicine, Changchun, China; 3 Northeast Asia Research Institute of Traditional Chinese Medicine, Changchun University of Chinese Medicine, Changchun, China

**Keywords:** time-restricted eating, healthy lifespan, non-communicable diseases, dietary pattern, metabolism

## Abstract

Time-restricted eating (TRE) is a dietary strategy that focuses on adjusting meal timing rather than adjusting diet structure or traditional caloric restriction, and has become a topic of interest in nutritional intervention research. Extending healthy lifespan is a major public health challenge. Diet is one of the key modifiable factors for preventing age-related diseases and maintaining overall health during the aging process. TRE has attracted widespread attention due to its advantages, such as high adherence and good safety, as well as its potential to improve metabolism. This narrative review retrieved clinical trials related to TRE from 2015 to 2025, comprehensively evaluating the latest advances in the field of healthy aging and analyzing the possible mechanisms of action. Research has shown that TRE has potential positive effects on the progression of age-related non-communicable diseases (NCDs), as well as cognitive and psychological functions. Some clinical trial results have shown that TRE can reduce body weight (3%−5%), improve glycated hemoglobin (0.3%−0.5%), and even partially lower total cholesterol (6%−7%) and other metabolic indicators. Meanwhile, we found that TRE exerts metabolic benefits depending on the coordinated regulation of “calorie restriction (CR)” and “eating time restriction.” These benefits are influenced by multiple factors, including TRE patterns (fasting duration and eating window), study populations (gender and metabolic status), and combined protocols (nutrition and exercise advice). Notably, in the current TRE studies, the TRE-8 (8-h daily eating duration) has received much attention, with existing evidence indicating its advantages in clinical applications. Similarly, the early eating window (eTRE), due to its alignment with circadian rhythms, also demonstrates relative advantages. In summary, in the field of healthy aging, TRE exerts a certain improvement effect on core indicators related to NCDs risk, such as weight control and glycolipid metabolism (fasting glucose and lipid profile). It also shows a trend of enhancing the quality of life and other aspects. However, its long-term safety, efficacy, and suitable populations require further validation through high-quality research.

## 1 Introduction

In recent decades, due to the continuous improvement of medical care, nutrition, healthcare, and public health measures, life expectancy around the world has significantly increased. At the same time, the prevalence of age-related non-communicable diseases (NCDs) has also increased accordingly (1), including diabetes, cardiovascular diseases (CVD), cancer, and neurodegenerative diseases. In terms of public health, NCDs have become the main diseases and the main cause of the medical economic burden. At the individual level, living longer does not mean living healthier or having a better quality of life (2). In recent years, the concept of “healthy lifespan” has attracted much attention. It is defined as “the process of cultivating and maintaining the functional abilities necessary for the health of the elderly,” with the core being to extend life expectancy while avoiding major chronic diseases (3). Therefore, the current assessment of healthy aging needs to take multiple dimensions into account. It should not only focus on core indicators related to the risk of chronic diseases, such as weight management and glucose and lipid metabolism, but also incorporate the assessment of internal capabilities, such as cognition and psychology, in order to comprehensively measure the health status of individuals during the aging process. There is no doubt that an unhealthy lifestyle has a negative impact on health status. Diet is the main adjustable factor for preventing NCDs and maintaining overall health in the process of aging. The implementation of scientific and effective dietary intervention strategies plays a key role in metabolic health, which can not only produce considerable social and economic benefits, but also promote the realization of the goal of “healthy lifespan” to a certain extent (4), thus improving overall wellbeing (5).

Time-Restricted Eating (TRE) is an important subtype of intermittent fasting, which is different from the existing diet strategies that focus on nutrients, such as the Mediterranean diet (MD) and calorie restriction (CR). TRE can exert its metabolic benefits without mandatorily requiring changes in dietary structure or calorie intake (6). It is this characteristic that makes the compliance of TRE generally higher than other diet patterns (7). With the advantages of convenient implementation, high patient adherence, and the ability to regulate metabolism across multiple systems, TRE has attracted much attention in the field of nutrition and has become a research hotspot for dietary intervention plans for NCDs. In recent years, many studies have suggested that TRE patterns may play a role in improving glycolipid metabolism disorders, reducing oxidative stress response, and optimizing the structure of intestinal flora. Its potential effects may involve regulating body weight, reducing the risk of chronic diseases, improving cognitive function and quality of life, thereby delaying the aging process, though no clear conclusions have been drawn yet (8).

The core mechanism of TRE metabolic benefits is still controversial. Although TRE does not require a mandatory calorie limit, some studies have observed that subjects may have a spontaneous calorie reduction of approximately 20%. Therefore, it remains unclear whether the metabolic benefits of TRE are mainly attributed to this spontaneous calorie reduction or the regulation of eating time independent of calorie restrictions. Meanwhile, the metabolic benefits of TRE are also affected by many factors, including the specific implementation protocols, fasting duration, and eating window selection, which may lead to differences in the relevant indicators of human metabolism. Most current studies adopt the classic “16:8 diet” (TRE-8) as the TRE intervention pattern, which can emphasize that the eating time needs to be synchronized with the circadian rhythm, allowing subjects to freely ingest energy within an 8-h daily eating window. This suggests that TRE-8 may be a relatively more prominent and advantageous pattern in current studies. However, its obvious advantages over other patterns have not been fully verified. Although existing studies have shown that TRE has the potential to reduce obesity risk and improve metabolic disorders, its long-term adherence, potential adverse effects, and suitable population range remain to be clarified through further studies.

In most TRE trials, the results combine the two effects of “calorie restriction” and “eating time restriction.” To distinguish the respective contributions of these two effects within TRE, this review will comprehensively compare and analyze the results of different intervention protocols, such as the difference between TRE and the control group under *ad libitum* conditions (results combine both effects), and focus on the TRE trials conducted under isocaloric conditions (outcomes may directly reflect the independent role of “eating time restriction”). This analytical strategy can provide clinical evidence for clarifying the mechanism of TRE. In summary, this review aims to evaluate the potential correlation between TRE and healthy lifespan. Based on the existing evidence, we focus on the role of TRE in obesity, diabetes, metabolic syndrome (MetS), non-alcoholic fatty liver disease (NAFLD), cancer, and neurodegenerative diseases. On this basis, we further explored its effectiveness, feasibility, and safety in the prevention of age-related NCDs, and then speculated on the possibility of promoting a healthy lifespan across the lifespan.

## 2 Methods

This study reviewed the latest research advances in TRE within the field of healthy aging by retrieving a large volume of TRE-related literature. We searched the PubMed database for articles published between 2015 and 2025. The search combined the key term “time-restricted eating” with each of the following: “health,” “aging,” “metabolism,” “non-communicable chronic diseases,” “obesity,” “weight management,” “diabetes,” “insulin,” “lipid metabolism,” “cardiovascular health,” “oxidative stress,” “inflammation,” “cancer,” “dementia,” and “cognitive function.” Inclusion criteria for research articles were as follows: (1) randomized controlled trials (RCTs) and non-randomized controlled trials involving human subjects; (2) inclusion of weight or glucose/lipid metabolism-related indicators; (3) published articles with full-text availability. Exclusion criteria applied to (1) cohort studies and observational studies; (2) fasting conducted as a religious observance (e.g., Ramadan or Seventh-day Adventist Sabbath). After careful review, 33 studies were ultimately selected for inclusion in Tables 1–**4** of this study. Since few clinical intervention trials have examined the effects of TRE on cancer or quality of life, observational studies were also included in the analysis for these two sections.

**Table 1:** Design, intervention protocol, and evaluation indicators for the time-restricted eating (TRE) trial in healthy participants.

**Type**	**Sample size and sex**	**Participants**	**Intervention group (protocol)**		**Control group (protocol)**		**Total calorie intake**	**Duration**	**Principal research outcomes**			**Reference**
			**Type of fasting**	**CR and NE**	**Comparison**	**CR and NE**			**Obesity indicators**	**Glycolipid indicators**	**Others**	
RCT	58 53 F 5M	Adults with obesity	TRE-6 (eat: 13:00–19:00) TRE-4 (eat: 15:00–19:00)	/	ND	/	**TRE-6 or TRE-4 (vs. ND)** ↓	10 weeks	**TRE-6 or TRE-4 (vs. ND)** ↓ W ↓ FM NS: VFM	**TRE-6 or TRE-4 (vs. ND)** ↓ FINS, ↓ HOMA-IR NS: FPG, HbA1c, LDL-C, HDL-C, TG	**TRE-6 or TRE-4 (vs. ND)** ↓ 8-isoPG NS: TNF-α, IL-6, SBP, DBP	(29)
RCT	139 68 F 71 M	Patients with obesity	TRE-8 (eat: 08:00–16:00)	CR: approximately −25% N	DCR	CR: approximately −25% N	**TRE-8 (vs. DCR)** NS	12 months	**TRE-8 (vs. DCR)** NS: W NS: WC, FM, LM, VFA	**TRE-8 (vs. DCR)** NS: GLU, 2hPPG, HOMA-IR, LDL-C, HDL-C, TC, TG	**TRE-8 (vs. DCR)** NS: SBP, DBP	(30)
RCT	90 74 F 16 M	BMI between 30 and 50 kg/m^2^ in adults	TRE-8 (eat: 12:00–20:00) Last 6 months (eat: 10:00–20:00)	N	DCR	CR: −25% N	**TRE-8 (vs. DCR)** NS	12 months	**TRE-8 (vs. DCR)** NS: W NS: WC, FM, LM, VFM, BMD	**TRE-8 (vs. DCR)** NS: FPG, FINS, HbA1c, HOMA-IR, LDL-C, HDL-C, TC, TG	**TRE-8 (vs. DCR)** NS: SBP, DBP	(31)
RCT	116 46 F 70 M	Adults with overweight and obesity	TRE-8 (eat: 12:00–20:00)	/	CMT	/	**TRE-8 (vs. CMT)** NS	12 weeks	**TRE-8 (vs. CMT)** NS: W NS: FM, LM	**TRE-8 (vs. CMT)** NS: FPG, FINS, HbA1c, HOMA-IR, LDL-C, HDL-C, TC, TG	**TRE-8 (vs. CMT)** NS: SBP, DBP	(32)
RCT	25 9 F 16 M	Healthy adults	TRE-8 TRE-4 (self-selected eating window)	/	ADF	/	**TRE-8 (vs. baseline)** NS **TRE-4 (vs. baseline)** NS **TRE-8 or TRE-4 (vs. ADF)** NS	8 weeks	**TRE-8 (vs. baseline)** NS: W NS: FM, SMM **TRE-4 (vs. baseline)** NS: W NS: FM, SMM **TRE-8 or TRE-4 (vs. ADF)** NS: W NS: FM, SMM	**TRE-8 (vs. baseline)** ↑ LDL-C, ↑ TC **TRE-4 (vs. baseline)** ↑ LDL-C **TRE-8 or TRE-4 (vs. ADF)** NS: LDL-C, HDL-C, TC, TG, GLU	**TRE-8 or TRE-4 (vs. baseline)** NS: CRP **TRE-8 or TRE-4 (vs. ADF)** NS: CRP	(33)
RCT	82 64 F 18 M	Healthy volunteers without obesity	eTRF-8 (eat: 06:00–15:00) mTRF-8 (eat: 11:00–20:00)	/	ND	/	**eTRE-8 (vs. mTRE-8)** NS **eTRE-8 or mTRE-8 (vs. ND)** ↓	5 weeks	**eTRE-8 (vs. mTRE-8)** NS: W NS: FM, BFP **eTRE-8 (vs. ND)** ↓ W ↓ FM, ↓ BFP	**eTRE-8 (vs. mTRE-8)** ↓ HOMA-IR NS: FPG, LDL-C, HDL-C, TC, TG **eTRE-8 (vs. ND)** ↓ HOMA-IR, ↓ FPG NS: LDL-C, HDL-C, TC, TG	**eTRE-8 (vs. mTRE-8)** NS: TNF-α, IL-8, hs-CPR, SBP, DBP **eTRE-8 (vs. ND)** ↓ TNF-α, ↓ IL-8 NS: hs-CPR, SBP, DBP	(34)
RCT	40 20 F 20 M	Healthy adults	TRE-8 (eat: 10:00–18:00) TRE-10 (eat: 09:00–19:00) TRE-12 (eat: 08:00–20:00)	N	ND	N	**TRE-8 (vs. TRE-10 or TRE-12 or ND)** NS	8 weeks	**TRE-8 (vs. TRE-10 or TRE-12 or ND)** ↓ W ↓ FM NS: VAT, LST	**TRE-8 (vs. TRE-10 or TRE-12 or ND)** NS: GLU, HOMA-IR, LDL-C, HDL-C, TC, TG	/	(35)
Single-arm	49 44 F 5 M	Healthy participants	TRE-8 (eat: 9:00–17:00)	N	/	/	**TRE-8 (vs. baseline)** NS	30 days	**TRE-8 (vs. baseline)** ↓ W ↓ BFP, MM	**/**	**TRE-8 (vs. baseline)** ↑ S1P ↑ prostaglandin-1	(36)
RCT	60 88%F 12%M	Adults with obesity	TRE-10 (Last meal time: 17:00–20:00)	CR: - 500–1,000 kcal/d N E: Daily walk	TRE-12 (Last meal time: 17:00–20:00)	CR: - 500–1,000 kcal/d N E: Daily walk	**TRE-10 (vs. TRE-12)** NS	8 weeks	**TRE-10 (vs. TRE-12)** ↓ W	**TRE-10 (vs. TRE-12)** NS: FPG	/	(38)
Random- ized pre-post	22 M	Physically active college-age men	TRE-8 (TRE 1: *Ad libitum* TRE 2: Isocaloric) (self-selected eating window)	TRE 2: stay within 300 kilocalories	/	/	**TRE-8 (vs. baseline)** NS **TRE-8 (TRE 1 vs. TRE 2)** NS	4 weeks	**TRE-8 (vs. baseline)** ↓ W ↓ FM	**TRE-8 (vs. baseline)** ↑ HDL-C NS: LDL-C, TC, TG	**TRE-8 (vs. baseline)** ↓ SBP, ↓ DBP NS: CRP, SOD	(39)
RCT	63 F	Middle-aged women BMI: ≥24	TRE-8 (eat: 10:00–18:00 or 12:00–20:00)	CR NE	no-TRE	CR NE	**TRE-8 (vs. no-TRE)** NS	8 weeks	**TRE-8 (vs. no-TRE)** ↓ W NS: WC, BFP, VAT	**TRE-8 (vs. no-TRE)** NS: FPG, FINS, HOMA-IR, LDL-C, HDL-C, TC, TG	**TRE-8 (vs. no-TRE)** NS: SBP ↓ DBP	(40)
non-RCT	32 F	Obese women BMI: ≥30	TRE-8 (eat: 12:00–20:00)	/	ND	/	/	3 months	**TRE-8 (vs. ND)** ↓ W ↓ WC, ↓ FM ↓ BFP, ↓ MM	**TRE-8 (vs. ND)** NS: FPG, FINS, HOMA-IR, LDL-C, HDL-C, TC, TG	**TRE-8 (vs. ND)** NS: SBP, DBP	(41)
Random- ized cross-over	34 F	Healthy middle-aged women	TRE-8	/	EXE	/	/	10 weeks	**Frist1 TRE-8 (within group)** ↓ W **EXE (within group)** NS: W **TRE-8 (vs. EXE)** ↓ W **Second2 TRE-8 (within group)** NS: W **EXE (within group)** NS: W **TRE-8 (vs. EXE)** NS: W	**Frist1 TRE-8 (within group)** ↓ FPG, ↓ TC **EXE (within group)** NS: FPG, TC **TRE-8 (vs. EXE)** ↓ FPG, ↓ TC **Second2 TRE-8 (within group)** NS: FPG, TC **EXE (within group)** NS: FPG, TC **TRE-8 (vs. EXE)** NS: FPG, TC	**Frist1 TRE-8 (within group)** NS: PSQI-K **Second2 TRE-8 (within group)** ↓ PSQI-K	(42)
RCT	108 57 F 51 M	Overweight older men and women	TRE-8 (eat: 12:00–20:00)	/	ND	/	/	6 weeks	**TRE-8 (vs. ND) Female** ↓ W NS: WC, VFM **Male** ↓ W ↓ WC, ↓ VFM	/	/	(43)
RCT	20	Healthy adults (>5 years of resistance training experience)	TRE-8 (eat: ~13:00–20:00)	Resistance	ND	Resistance	**TRE-8 (vs. ND)** ↓	12 months	**TRE-8 (vs. ND)** ↓ W ↓ FM NS: VAT	**TRE-8 (vs. ND)** ↓ GLU, ↓ Insulin ↓ HOMA-IR ↓ LDL-C, ↓ TG ↑ HDL-C NS: TC	**TRE-8 (vs. ND)** ↓ TNF-α ↓ IL-1β, ↓ IL-6	(44)
RCT	131 F	Women with overweight/obesity	1.TRE ( ≤ 10-h daily eating window) 2.HIIT 3.TRE+HIIT ( ≤ 10-h daily eating window)	/	CON	/	**TRE or TRE+HIIT (vs. CON)** ↓	7 weeks	**TRE or HIIT or TRE+HIIT (vs. CON)** ↓ W ↓ VFA	**TRE+HIIT (vs. CON)** ↓ HbA1c, ↓ HDL-C NS: LDL-C, TC, TG **TRE or HIIT (vs. CON)** NS: HbA1c NS: HDL-C, LDL-C, TC, TG	**TRE or HIIT or TRE+HIIT (vs. CON)** NS: SBP, DBP	(45)

F, FEMALE; M, male; health adults, age ≥18; no history of cardiovascular disease, metabolic disease, or gastrointestinal disease; not undergoing other fat loss measures (e.g., medications or surgery); and not pregnant or breastfeeding; TRE-x, eating within × hours, with the rest of the time being a fasting period; e-TRE, early eating window; m-TRE, middle eating window; HIIT, high-intensity interval training; CR, caloric restriction; N, nutrition recommendations; E, exercise recommendations; ND, normal diet; EXE, 14-h time-extended eating; CMT, continuous meal timing; ADF, fast every other day for 24 h, and eat freely on non-fasting days; NS, no significance; W, weight; WC, waist circumference; FM, fat mass; LM, lean mass; LST, lean soft tissue; BFP, body fat percentage; SMM, skeletal muscle mass; BMD, bone mineral density; MM, muscle mass; VFM, visceral fat mass; VAT, visceral adipose tissue; VFA, visceral fat area; GLU, glucose level; FPG, fasting blood glucose; HOMA-IR, homeostatic model assessment of insulin resistance; 2hPPG, 2-h postprandial glucose; FINS, fasting insulin; HbA1c, glycated hemoglobin; LDL-C, low-density lipoprotein cholesterol; HDL-C, high-density lipoprotein cholesterol; TC, total cholesterol; TG, triglycerides; 8-isoPG, 8-isoprostane; SBP, systolic blood pressure; DBP, diastolic blood pressure; CRP, C-reactive protein; hs-CRP, high-sensitivity C-reactive protein; TNF-α, tumor necrosis factor alpha; SOD, superoxide dismutase; S1P, sphingosine-1-phosphate; IL-1β, interleukin 1 beta; IL-8, interleukin 8; IL-6, interleukin 6; PSQI-K, Pittsburgh sleep quality index – Korean Version.

Intervention group (vs. Control group): Comparison results of indicators between the intervention group and the control group.

## 3 Health promotion mechanisms associated with TRE

**Figure 1:**
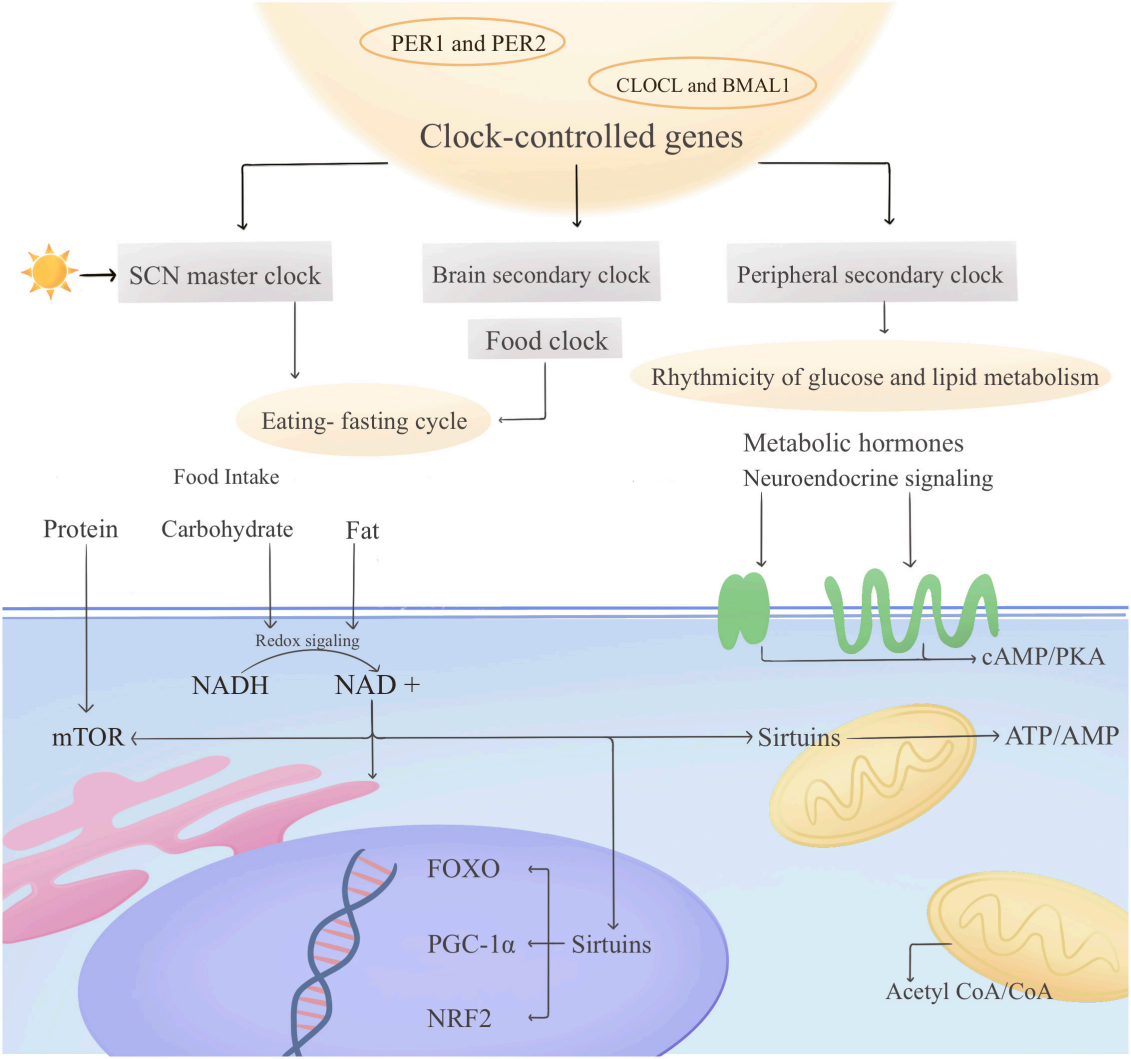
The main health promotion mechanisms related to TRE.

Currently, the health-promoting mechanisms of TRE are believed to primarily involve the activation of fasting physiology and the synchronization of peripheral and central circadian rhythms (Figure 1). Additionally, it reduces oxidative stress and improves gut microbiota composition. Therefore, this section will review the primary mechanisms potentially involved in TRE, while other detailed potential pathways require further exploration. It is important to note that the health benefits of TRE are co-mediated by “caloric restriction” and “eating time restriction,” whereas the health-promoting mechanisms activated by these two factors exhibit certain disparities. The shared mechanism between TRE and traditional CR is “calorie restriction,” which activates metabolic repair pathways (e.g., the fasting physiology). Essentially, this corresponds to the spontaneous reduction in caloric intake observed with TRE. The core mechanism unique to TRE is “eating time restriction,” which synchronizes physiology with circadian rhythms through fixed eating windows, independent of “calorie restriction.” Additionally, TRE reduces oxidative stress and improves intestinal microbial structure.

### 3.1 Synchronize the peripheral and central body clocks

TRE restricts the daily eating window to the period when the circadian rhythm is active, reconstructing the phase synchronization between peripheral organs and the central clock of the suprachiasmatic nucleus (SCN), thereby correcting the phase dysregulation caused by modern lifestyles (9). Specifically, the mechanism of this process lies in the fact that TRE directly acts on peripheral organs through regular feeding signals (such as the periodic fluctuations of insulin and free fatty acids), activating silent information regulator-1 (Sirtuin-1 and Sirt1) (10), and then enhancing the rhythmic expression of core clock genes such as BMAL1 and PER2, effectively correcting the phase delay of the peripheral biological clock relative to the SCN. At the same time, by coordinating the feeding–fasting cycle with the biological clock signals, TRE can reverse the genes involved in glucose imbalance in the liver (such as GLUT2, pyruvate kinase, glucokinase, and glycogen synthase) and the genes involved in lipid imbalance (such as acetyl-CoA carboxylase, diacylglycerol O-acyltransferase, and medium-chain acyl-CoA dehydrogenase) (11, 12). Generally speaking, TRE achieves the global coordination of the metabolic functions between the peripheral and central biological clock systems, restoring the temporal precision of the expression of metabolic genes.

### 3.2 Activate fasting physiology

TRE triggers a switch of metabolic fuels by extending the fasting period, enabling the body to shift from relying on glucose to fatty acid oxidation and ketone body production (13). After the fasting time reaches 12–16 h, the liver glycogen reserves are depleted, forcing the body to mobilize fat and activate the β-oxidation pathway, generating acetyl-CoA and synthesizing ketone bodies. Similarly, this process activates the AMPK and mTOR signaling pathways, thereby promoting fatty acid β-oxidation and the autophagy process (14). The increased ketone bodies not only serve as an alternative energy source but also regulate gene expression, DNA repair, and genomic stability by inhibiting histone deacetylase (HDAC). Simultaneously, the reduction in the ATP/ADP ratio activates the AMPK/mTORC1 pathway, enhancing autophagy activity and promoting catabolism (13). This optimizes mitochondrial function and accelerates the clearance of metabolic waste products, such as damaged proteins and abnormal mitochondria (15, 16). The enhancement of mitochondrial function is achieved through the biosynthesis mediated by Forkhead Box Protein O, Peroxisome Proliferator-Activated Receptor-γ Coactivator 1-α (PGC-1α), and Nuclear Factor Erythroid 2-Related Factor 2 (NRF2). This process increases the oxygen consumption of individual mitochondria by approximately 40%, significantly improving the efficiency of energy metabolism. In conclusion, by enhancing mitochondrial function and metabolic flexibility, TRE further optimizes energy metabolism, enabling the body to switch fuel sources more efficiently under different energy demands, thereby achieving the benefits of metabolic regulation.

### 3.3 Reduce oxidative stress response

TRE significantly improves oxidative stress and inflammatory responses through a multi-dimensional regulatory mechanism. At the level of antioxidant defense, TRE forms a dual protective barrier by activating the AMPK signaling pathway (17). On the one hand, it inhibits the excessive production of mitochondrial reactive oxygen species (ROS), and on the other hand, it upregulates the expression of key antioxidant enzymes such as superoxide dismutase (SOD) and glutathione peroxidase (18). It is worth noting that the autophagy process induced by fasting plays a synergistic role in this process. By removing dysfunctional organelles, it effectively blocks the continuous accumulation of ROS, thereby reducing the level of oxidative stress. In addition, studies have shown that TRE can also reduce the expression of inflammatory mediators such as interleukin-6 (IL-6) and tumor necrosis factor-α (TNF-α) in adipose tissue, and significantly inhibit the activity of NF-κB, blocking the cascade amplification of inflammatory signals from the source (19). More importantly, the increase in adiponectin levels induced by TRE activates the PI3K/Akt signaling pathway, reducing the release of vascular endothelial cell adhesion molecules (such as VCAM-1, ELAM-1, and ICAM-1), thereby inhibiting local and systemic inflammatory responses (20).

### 3.4 Improve intestinal microbial structure

TRE restores the chronobiological characteristics of the microbial community through regular feeding times (21), mainly manifested in improving the structure of gut microbiota (increasing the number of beneficial bacteria while reducing the number of harmful bacteria), thus improving age-related gut barrier dysfunction. Specifically, the TRE intervention involving a 16-h daily fast resulted in reduced abundance of potentially pro-inflammatory bacteria such as *Ruminococcaceae* and *Alistipes*, while significantly increasing the abundance of *Akkermansia muciniphila*, which possesses mucosal protective functions (22). At the same time, the proportions of bacteria closely related to energy metabolism, such as *Ruminococcus* and *Coprococcus* spp., increased (23). Additionally, TRE significantly increases the abundance of probiotics such as *Bifidobacteria* and *Lactobacillus*, promoting the proliferation of specific metabolically active genera within the phylum Firmicutes, notably short-chain fatty acid (SCFA)-producing bacteria like *Clostridium butyricum* and *Roseburia*. These metabolically active bacteria generate short-chain fatty acids (SCFAs) such as acetic acid, propionic acid, and butyric acid by fermenting dietary fiber, exerting anti-inflammatory effects, enhancing gut barrier function, and reducing gut permeability (24). It is noteworthy that TRE plays a particularly crucial role in promoting butyrate-producing bacteria, such as certain *Clostridium* species, *Blautia*, and *Eubacterium*. At present, the function of these bacteria is closely related to the improvement of cognitive function, the delay of aging, and anti-inflammatory effects (25).

## 4 The effects of TRE on NCDs and healthy lifespan

**Figure 2:**
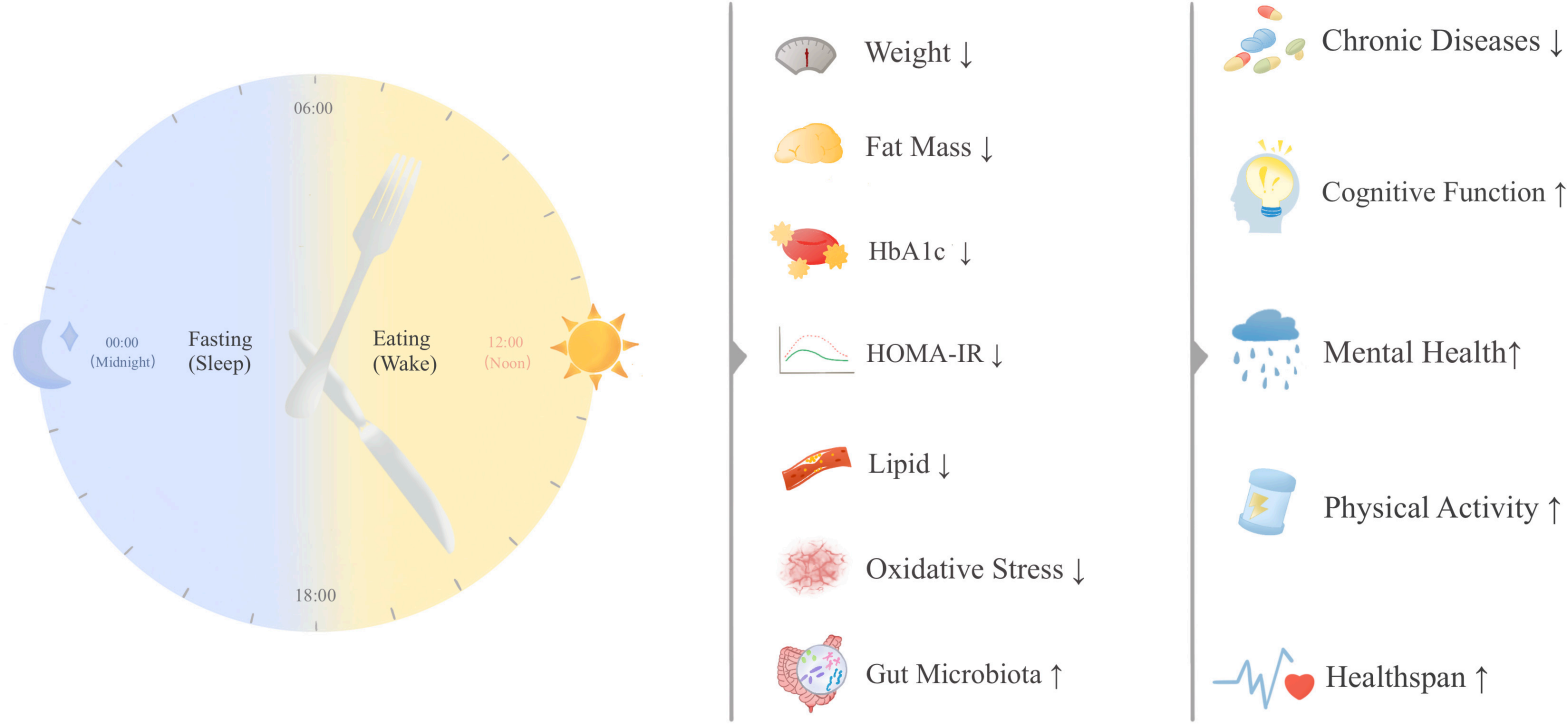
Time-restricted eating and healthy aging.

As a dietary pattern, TRE exerts positive effects on extending healthy lifespan, offering a novel intervention strategy. Current research on TRE primarily focuses on overweight, obesity, prediabetes, diabetes, MetS, NAFLD, and cancer. This article reviews the clinical evidence for TRE, indicating its potential to facilitate weight reduction, improve insulin resistance, and ameliorate dyslipidemia. To accurately assess its role in healthy aging, we primarily focus on risk indicators for NCDs onset/progression (including weight, blood glucose, and lipids) while also considering assessments of intrinsic capacity (including cognitive and mental health and quality of life) (26). These findings collectively support TRE's potential to enhance overall health (Figure 2). Additionally, certain adverse effects of TRE are discussed.

### 4.1 Weight and body composition

The weight trajectory can serve as a marker of the long-term energy balance status at different stages of the life cycle. During the aging process, as individuals advance in age (with middle age being a critical turning point), metabolic capacity often declines, leading to an increased risk of obesity. Preclinical studies have confirmed that implementing time-restricted feeding (TRF) may partially reverse abnormal circadian rhythmic oscillations of clock genes and their target genes, as well as the dysregulation in expression or activity of metabolic regulatory proteins such as AMPK, mTOR, and CREB that frequently accompany these changes (27). Therefore, TRE can be considered a potential intervention strategy for regulating energy metabolism (28). We summarize the trial designs, intervention protocols, and principal research outcomes of TRE trials involving healthy participants without a history of chronic or major diseases (Table 1).

Clinical research on TRE in some healthy populations suggests that it exerts certain positive effects on weight and glycolipid metabolism-related indicators. However, its metabolic benefits are influenced by multiple factors, including calorie restriction, fasting duration, eating time windows, participant gender, exercise regimens, and others. First, we observed that the reduction in calorie intake may be an important reason why TRE plays a role. For example, a 10-week clinical trial showed that both TRE-4 and TRE-6 protocols spontaneously reduced calorie intake while achieving mild weight loss (~3%), accompanied by a decrease in insulin resistance and oxidative stress. In addition, there was no significant change in the dietary quality (such as sugar and alcohol intake) during the period (29). It should be noted that some long-term studies have also not observed significant advantages in weight loss and body composition improvement (e.g., body fat reduction) compared with CR. Specifically, two 12-month RCTs showed that the TRE-8 protocols (with either ~25% calorie restriction or *ad libitum* eating) did not produce a more significant weight loss effect than the daily calorie restriction (DCR) diets, suggesting calorie restriction may be the primary mechanism underlying TRE's benefits (30, 31). Similarly, compared with conventional meal timing (CMT), a 12-week TRE-8 (12:00–20:00) intervention did not reduce calorie intake, and there was no significant difference in weight loss, fasting plasma glucose (FPG), glycated hemoglobin (HbA1C), lipid profiles, or blood pressure (32). Another TRE-4 or TRE-8 (self-selected eating window) also confirmed this point. Despite no difference in caloric intake before and after intervention, TRE showed no significant weight change or positive metabolic benefits related to blood glucose and lipids. Instead, it raised low-density lipoprotein cholesterol (LDL-C) levels (33). These studies suggest that the metabolic benefits of TRE may mainly come from “calorie restriction.” Additionally, research indicates that under caloric reduction, different eating time windows can vary in metabolic effects. Specifically, an RCT study confirmed that compared with mTRF-8 (12:00–20:00), eTRF-8 (06:00–15:00) has a more significant effect on improving insulin resistance in non-obese individuals, accompanied by increased diversity of gut microbiota and enhanced clock gene rhythms. These changes cannot be explained solely by weight loss (34).

Subsequently, we observed that some current clinical intervention trials have excluded the “calorie restriction” factor. Significant changes in some clinical indicators were still observed when TRE-8 did not cause changes in calorie intake. For example, an 8-week RCT showed that TRE-8 can still improve body composition without reducing calorie intake. No significant changes were observed in the TRE-10/12 pattern with shorter fasting durations, which suggests that TRE-8 is a more effective TRE intervention pattern (35). Moreover, some studies have confirmed that the TRE-8 intervention can exert certain positive effects on aging-related factors. Specifically, after 30 days of TRE-8 intervention, sustained weight loss of 2%−4% was observed even without caloric restriction. Peripheral blood showed reduced proportions of CD4+ senescent T cells, alongside significant upregulation of serum metabolites with anti-inflammatory and anti-aging effects [e.g., sphingosine 1-phosphate (S1P) and prostaglandin-1]. Concurrently, the abundance of longevity-associated gut microbiota (such as *Akkermansia* and *Rikenellaceae*) also increased (36). This is consistent with existing research evidence. TRE may exert metabolic benefits independent of caloric restriction by regulating gut microbial composition through improving the circadian rhythms of gut microbes (37). At the same time, studies on other patterns of TRE further suggest that “differences in fasting duration may explain variations in TRE efficacy.” For example, in the study of TRE with the commercial weight loss program (Jenny Craig^®^ Rapid Results™), both the TRE-10 and TRE-12 groups carried out calorie restriction and macronutrient adjustment. The results showed that the TRE-10 group significantly lost weight (38). Overall, these findings indicate that the health benefits of TRE depend not only on the reduction of calories but also on directly regulating the metabolic rhythm through restricted eating windows (such as optimizing lipid metabolism). The widely studied TRE-8 pattern may have relative advantages in metabolic regulation, indicating that appropriately extending fasting durations could enhance intervention efficacy.

After analyzing multiple TRE-8-related studies in healthy populations, we noticed that the metabolic benefits of TRE may tend to be gender-different. We observed that although there was no obvious spontaneous decrease in calorie intake in TRE, the research results showed that some index results still had a statistically significant improvement. Specifically, in physically active male university-aged subjects, a 4-week TRE-8 intervention (self-selected eating window) achieved reductions in fat mass (FM), increases in high-density lipoprotein cholesterol (HDL-C), and decreases in blood pressure, under isocaloric conditions (39). In an 8-week RCT study of middle-aged women, it was also found that, under identical calorie restriction conditions, TRE-8 significantly reduced body weight, body mass index (BMI), and diastolic blood pressure (DBP) more effectively than the non-TRE approach. It is worth noting that this method may increase FPG and worsen insulin resistance (40). However, some trials did not exclude the “calorie restriction” factor. For example, a 3-month non-RCT showed that the weight, waist circumference (WC), and body fat percentage (BFP) of obese women decreased significantly after TRE-8 intervention, while there was no change in the indicators related to glycolipid metabolism (FPG, fasting insulin, and lipid profile). In addition, the 30-year risk of cardiovascular disease (CVDRisk30y) was reduced by 12%, with a positive correlation with BFP and a negative correlation with muscle percentage (41). Similar to this research finding, an RCT reported that compared with the extended eating window group (EXE), female participants also achieved significant weight loss through the TRE-8 intervention (42). At the same time, a 6-week TRE-8 intervention RCT showed a significant decrease in the weight of both elderly men and women by 1.8 and 1.3 kg, respectively. Notably, elderly men exhibited significant reductions in visceral fat mass and WC, whereas these measures showed no significant change in elderly women (43). Therefore, there may be relatively few studies on male subjects, but overall, the available evidence still shows many benefits, such as reducing weight, body fat, and improving lipid metabolism indicators. Although women in some studies have also reduced their weight and WC, the improvement of sugar-lipid metabolism-related indicators is still limited.

In addition, in the absence of other interventions (such as physical exercise), TRE tends to produce slight improvements in blood lipid levels of healthy individuals. We noticed that in a TRE-8 combined resistance training trial, the blood lipid spectrum (LDL-C and HDL-C) was significantly improved after 12 months of intervention. The study included healthy adults with at least 5 years of resistance training experience. The TRE group and the normal diet (ND) group were compared, with both groups combined with resistance training. Results showed reduced weight, fat mass, insulin-like growth factor 1, and testosterone levels in the TRE group. Additionally, the group's inflammatory markers [interleukin-6 (IL-6) and interleukin-1β (IL-1β), tumor necrosis factor-α (TNF-α)] and insulin sensitivity (glucose, insulin, and HOMA-IR) were significantly improved, with no impact on muscle performance. Notably, this long-term TRE trial reduced daily caloric intake by approximately 10%, with the reduction primarily derived from carbohydrates and fats while protein intake remained unchanged (44). Similarly, a study of TRE combined with high-intensity intermittent training (HIIT) showed that TRE+HIIT offers metabolic advantages for overweight/obese women, primarily reflected in improved body composition (fat mass) and long-term blood glucose control (HbA1c) (45). In short, in combination with some exercise interventions, TRE may have better metabolic benefits. However, it is necessary to pay attention to adaptive changes in body composition. Although this strategy does not impose strict calorie restriction, spontaneous calorie reduction is often associated with lean mass loss, potentially linked to protein conversion imbalance and water changes. While no severe adverse effects from lean mass reduction have been reported, existing researches recommend combining TRE with resistance training and adequate protein supplementation to ensure sustained weight and fat loss.

### 4.2 Pre-diabetes and diabetes

With increasing age, pancreatic β-cell function undergoes progressive decline, leading to an imbalance in blood glucose regulation—a core pathological basis for the high prevalence of diabetes. Meanwhile, long-term irregular eating patterns and continuous 24-h eating behavior trigger circadian rhythm disruption, which in turn interferes with the rhythmic secretion of insulin and directly contributes to insulin resistance (46). This metabolic imbalance further disrupts the rhythmic dynamic balance between hepatic glucose output and peripheral tissue glucose uptake. Additionally, disturbances in glucose metabolism, such as elevated blood glucose and persistent insulin resistance, act as key mediators driving aging and the progression of other chronic diseases by inducing mitochondrial dysfunction and oxidative stress. Current research suggests that TRE may exert a corrective effect on circadian dysregulation of glucose metabolism by synchronizing the eating window with the circadian rhythm (47). Its potential mechanisms of action may involve multiple pathways, including activation of the AMPK signaling pathway and regulation of GLUT4 translocation. This suggests that TRE may influence insulin resistance reduction and blood glucose regulation, so we have summarized the trial designs, intervention protocols, and principal research outcomes of TRE trials involving prediabetes/diabetes participants (Table 2). Multiple clinical data show that TRE has differences in improving core indicators (weight, HbA1c, etc.) for prediabetic/diabetic patients due to differences in calorie intake, study population, program design, and other factors.

**Table 2:** Design, intervention protocol, and evaluation indicators for the time-restricted eating (TRE) trial in participants with prediabetes/diabetes.

**Type**	**Sample size and sex**	**Participants**	**Intervention group (protocol)**		**Control group (protocol)**		**Total calorie intake**	**Duration**	**Principal research outcomes**			**Reference**
			**Type of fasting**	**CR and NE**	**Comparison**	**CR and NE**			**Obesity indicators**	**Glycolipid indicators**	**Others**	
RCT	51 21 F 30 M	T2D and overweight/obesity	TRE-9 (eat: 10:00–19:00)	/	DIET	/	**TRE-9 (vs. DIET)** NS **TRE-9 (vs. baseline)** ↓	6 months	**TRE-9 (vs. DIET)** NS: W NS: FM, BFP, VAT, LM	**TRE-9 (vs. DIET)** NS: FINS, HbA1c, HOMA-IR	**TRE-9 (vs. DIET)** NS: SBP, DBP	(48)
RCT	75 53 F 22 M	T2D adults	1.TRE-8 (eat: 12:00–20:00) 2.DCR	1.TRE-8 CR:/ N: DIET 2.DCR CR:−25% N: DIET	ND	CR:/ N: DIET	**TRE-8 (vs. ND)** ↓	6 months	**TRE-8 (vs. ND)** ↓ W ↓ FM, ↓ WC **TRE-8 (vs. DCR)** NS: W NS: FM, WC	**TRE-8 (vs. ND)** ↓ GLU, ↓ HbA1c NS: LDL-C, HDL-C, TC, TG **TRE-8 (vs. DCR)** NS: GLU, HbA1c, LDL-C, HDL-C, TC, TG	**TRE-8 (vs. ND)** NS: SBP, DBP **TRE-8 (vs. DCR)** NS: SBP, DBP	(49)
RCT	120 55 F 65 M	Overweight T2D adults	TRE-10 (eat: 8:00–18:00)	/	ND	/	**TRE-10 (vs. ND)** ↓	14 weeks	**TRE-10 (vs. ND)** ↓ W	**TRE-10 (vs. ND)** ↓ FPG, ↓ HbA1c ↓ HOMA-IR ↓ LDL-C, ↓ TG, ↓ TC NS: HDL-C	/	(51)
RCT	100 66 F 34 M	Overweight, prediabetes, or obesity (High risk of T2D)	TRE-10 (self-selected eating window)	N	ND	N	**TRE-10 (vs. ND)** NS	3 months	**TRE-10 (vs. ND)** NS: W NS: FM	**TRE-10 (vs. ND)** NS: FPG, HbA1c, LDL-C, HDL-C, TC, TG	**TRE-10 (vs. ND)** NS: SBP, DBP	(52)
RCT	72 50 F 22 M	IFG adults	TRE-9 (eat: 8:00–17:00)	/	ND (usual care)	/	**TRE-9 (Compliance) (vs. ND)** ↓	12 weeks	**TRE-9 (Compliance) (vs. ND)** NS: W	**TRE-9 (Compliance) (vs. ND)** ↓ FPG, ↓ FINS ↓ HbA1c, ↓ HOMA-IR ↓ TG NS: LDL-C, HDL-C, TC	**TRE-9 (Compliance) (vs. ND)** ↓ DBP NS: hs-CRP, SBP	(53)
RCT	27 17 F 10 M	Adolescents with obesity and new-onset T2D	lTRE-8 (eat: 12:00–20:00)	N	ND	N	**TRE-8 (vs. ND)** ↓	3 months	**TRE-8 (vs. ND)** NS: W	**TRE-8 (vs. ND)** NS: HbA1c, LDL-C, HDL-C, TG	**TRE-8 (vs. ND)** NS: ALT	(54)
Random- ized cross-over	8 M	Overweight and obese with prediabetes	eTRE-6 (isocaloric) (Last meal time: before 15:00)	N	ND (isocaloric) 3 meals in 12 hs	N	**eTRE-6 (vs. ND)** NS	5 weeks	**eTRE-6 (vs. ND)** NS: W	**eTRE-6 (vs. ND)** ↓ FINS NS: FPG, LDL-C, HDL-C	**eTRE-6 (vs. ND)** ↓ 8-isoPG ↓ SBP, ↓ DBP NS: hs-CRP, IL-6	(55)

F, female; M, male; T2D, type 2 diabetes; IFG, impaired fasting glucose; TRE-x, eating within × hours, with the rest of the time being a fasting period; e-TRE, early eating window; l-TRE, late eating window; DIET, Based on the Public nutrition guidelines for patients with type 2 diabetes; CR, caloric restriction; DCR, daily caloric restriction; ND, normal diet; N, nutrition recommendations; W, weight; WC, waist circumference; FM, fat mass; LM, lean mass; BFP, body fat percentage; VAT, visceral adipose tissue; FPG, fasting blood glucose; GLU, glucose level; FINS, fasting insulin; HbA1c, glycated hemoglobin; HOMA-IR, homeostatic model assessment of insulin resistance; LDL-C, low-density lipoprotein cholesterol; HDL-C Cho, High-Density Lipoprotein cholesterol; TC, total cholesterol; TG, triglycerides; SBP, systolic blood pressure; DBP, diastolic blood pressure; hs-CRP, high-sensitivity C-reactive protein; 8-isoPG, 8-isoprostane; IL-6, interleukin 6; ALT, serum alanine aminotransferase; ALT, serum alanine aminotransferase.

Intervention group (vs. Control group): Comparison of the group's core intervention with its baseline or other interventions.

After analyzing some research results, we found that the metabolic benefits of TRE may be directly related to “calorie restriction.” For example, a 6-month RCT involving patients with type 2 diabetes (T2D) showed that, with reduced calorie intake, the TRE-9 regimen decreased indicators such as body weight, body fat, and insulin resistance index. Furthermore, it achieved a reduction in HbA1c that was comparable to that of the individualized diabetes nutrition guidelines (DIET) group, while demonstrating higher adherence. These findings suggest TRE may serve as an alternative dietary strategy to DIET for improving glycemic control in T2D patients (48). Similarly, several trials demonstrated that “significant calorie reduction” amplifies TRE's metabolic benefits. For instance, a 6-month TRE-8 study showed the TRE-8 group achieved a greater daily calorie reduction (313 kcal) than the DCR group (197 kcal), resulting in superior weight loss (−3.6%) compared to DCR (−1.8%). However, both groups exhibited similar reductions in glycated hemoglobin (HbA1c) levels (−0.91 and −0.94%, respectively), which may be related to both groups receiving individualized diabetes nutrition counseling that has been proven to improve HbA1c levels in patients with T2D. Furthermore, TRE groups demonstrated higher adherence compared to DCR groups (49, 50). Another 14-week RCT also showed that compared with ND, TRE-10 reduced the spontaneous calorie intake of participants, which significantly improved the weight and glycolipid-related indicators of overweight T2D patients (51). Interestingly, a study showed that a 3-month TRE-10 intervention did not affect calorie intake, and no improvements related to weight loss and glycolipid metabolism were observed (52). Therefore, we speculate that the effects of TRE are likely to be related to the degree of realization of “caloric restriction.” Notably, research confirmed TRE may confer metabolic benefits independent of weight loss. Specifically, an RCT involving impaired fasting glucose (IFG) patients demonstrated that adhering to the TRE-9 protocol, with reduced calorie intake, significantly improved FPG, HbA1c, fasting insulin, HOMA-IR, triglycerides (TG), and DBP levels, even without weight loss (53). Furthermore, there are also feasibility studies of TRE interventions targeting adolescents with T2D, which adopted an lTRE (12:00–20:00) protocol. However, compared with the control group, no significant metabolic benefits were observed, which may be due to the small sample size or impaired pancreatic islet function in adolescents (54). In summary, TRE research findings indicate that the effects on the aforementioned trial principal research outcomes exhibit a certain degree of synchrony with differences in caloric intake. This suggests that the improvement effect of TRE on relevant metabolic indicators may be more significantly related to “calorie restriction.”

Of course, there are also research results suggesting that TRE may bring metabolic benefits independent of “calorie restriction.” Whether TRE interventions can yield comparable metabolic benefits under isocaloric conditions has drawn attention. For instance, a 5-week randomized crossover trial of eTRE-6 (early eating window) in men with prediabetes minimized confounding effects from food intake or meal frequency variations by strictly requiring participants to consume only researcher-provided meals. Results showed that even without caloric reduction or weight loss, TRE still significantly improved participants' insulin levels, insulin sensitivity, β-cell responsiveness, blood pressure, and oxidative stress levels, suggesting it may function in addition to caloric restriction or weight loss. However, there were no significant improvements in blood glucose levels, arterial stiffness, lipid profiles, and inflammatory markers of the participants (55). Therefore, short-term TRE can improve the impaired state of pancreatic islet function, but the study needs to be further verified due to the small sample size and single gender (all men).

### 4.3 MetS and NAFLD

In the context of the accelerating global aging process, metabolic disorders can be regarded as a core threat to public health. Significantly, the complex manifestations are collectively represented by MetS, which encompasses components such as central obesity, impaired glucose tolerance, dyslipidemia, and hypertension. At present, NAFLD is generally considered to be the pathological outcome of MetS at the liver level (56). Specifically, the relationship between the two depends on the common pathological mechanism, covering insulin resistance, lipotoxic lipid release, increased liver fat production, and systemic inflammation activation (57, 58). Preclinical studies suggested that TRF has the potential to improve the pathological status of MetS and NAFLD (59). Under isocaloric conditions, TRF intervention resulted in reduced hepatic inflammation without significant weight loss. These benefits may be mediated through mechanisms such as reducing lobular hepatic inflammation and reducing endoplasmic reticulum stress levels (60). Therefore, TRE is considered likely to delay the development of MetS and NAFLD. We summarize the trial designs, intervention protocols, and principal research outcomes of TRE trials for MetS and NAFLD participants (Table 3).

**Table 3:** Design, intervention protocol, and evaluation indicators for the time-restricted eating (TRE) trial in MetS/NAFLD participants.

**Type**	**Sample size and sex**	**Participants**	**Intervention group (protocol)**		**Control group (protocol)**		**Total calorie intake**	**Duration**	**Principal research outcomes**			**Reference**
			**Type of fasting**	**CR and NE**	**Comparison**	**CR and NE**			**Obesity indicators**	**Glycolipid indicators**	**Others**	
Single-arm	19 6 F 13 M	MetS adults	TRE-10 (self-selected eating window)	/	/	/	**TRE-10 (vs. baseline)** ↓	12 weeks	**TRE-10 (vs. baseline)** ↓ W ↓ WC	**TRE-10 (vs. baseline)** ↓ LDL-C, ↓ TC NS: FPG, FINS, HbA1c, HOMA-IR, HDL-C, TG	**TRE-10 (vs. baseline)** ↓ SBP, ↓ DBP NS: hs-CRP, ALT, AST NS: PSQI	(61)
RCT	108 56 F 52 M	MetS adults	Personalization TRE (ended ≥3 h before habitual bedtime)	SOC N	SOC	SOC N	**TRE (vs. baseline)** −350 kcal/d	3 months	**TRE (vs. SOC)** ↓ W ↓ FM, ↓ BFP NS: LM	**TRE (vs. SOC)** ↓ HbA1c ↓ LDL-C NS: FPG, FINS, HOMA-IR, HDL-C	**TRE (vs. SOC)** NS: hs-CRP, SBP, DBP	(62)
RCT	162 60 F 102 M	MetS adults	TRE-8 or TRE-8+LCD (eat: 8:00–16:00 or 12:00–20:00)	N	LCD	N	/	3 months	**TRE-8 or TRE-8+LCD (vs. baseline)** ↓ W ↓ WC	**TRE-8 or TRE-8+LCD (vs. baseline)** ↓ FINS, ↓ HOMA-IR ↓ TG	**TRE-8 or TRE-8+LCD (vs. baseline)** ↓ UA	(63)
RCT	88 39 F 49 M	Adults with obesity and NAFLD	TRE-8 (eat: 08:00–16:00)	CR N	DCR	CR N	**TRE-8 (vs. baseline)** **↓TRE-8 (vs. DCR)** NS	12 months	**TRE-8 (vs. baseline)** ↓ W ↓ WC, ↓ FM ↓ VFA **TRE-8 (vs. DCR)** NS: W NS: WC, FM, VFA	**TRE-8 (vs. baseline)** ↓ IHTG ↓ FPG, ↓ HbA1c ↓ HOMA-IR, ↓ LDL-C ↑ HDL-C, ↓ TC ↓ TG **TRE-8 (vs. DCR)** ↓ HOMA-IR NS: FPG, HbA1c, LDL-C, HDL-C, TC, TG, IHTG	**TRE-8 (vs. baseline)** ↓ LS ↓ ALT, ↓ AST, ↓ GGT ↓ SBP, ↓ DBP **TRE-8 (vs. DCR)** NS: LS, ALT, AST, GGT, SBP, DBP	(64)
Random- ized Cross-Over	32 13 F 19 M	NAFLD adults	TRE-8 (eat: 12:00–20:00)	/	SOC	CR NE (Aerobic and Resistance)	**TRE-8 (vs. SOC)** NS	12 weeks	**TRE-8 (vs. SOC)** ↓W ↓WC	**TRE-8 (vs. SOC)** NS: FPG, Insulin, HOMA-IR, LDL-C, TC, TG	**TRE-8 (vs. SOC)** ↓ CAP NS: SBP, DBP NS: LS	(65)
RCT	45 18 F 27 M	Patients with NAFLD and overweight or obesity	TRE-8 (self-selected eating window) (Isocaloric)	N Low-sugar diet (WHO)	ND (Isocaloric = TRE)	N	**TRE-8 (vs. ND)** NS	12 weeks	**TRE-8 (vs. ND)** ↓ W ↓ WC, ↓ FM	**TRE-8 (vs. ND)** ↓ FPG ↓ LDL-C, ↓ TC, ↓ TG NS: Insulin, HOMA-IR, HDL-C	**TRE-8 (vs. ND)** ↓ CAP, ↓ hs-CRP ↓ ALT, ↓ AST ↓ GGT	(67)
RCT	42 16 F 26 M	Overweight and obese patients diagnosed with MAFLD	TRE-8 (self-selected eating window)	N LOV	ND	N	**TRE-8 (vs. ND)** NS	12 weeks	**TRE-8 (vs. ND)** ↓ W ↓ WC	**TRE-8 (vs. ND)** ↓ Insulin, ↓ TG ↑ HDL-C NS: TC, LDL-C	**TRE-8 (vs. ND)** ↓FLI ↓TNF-α ↓ALT, ↓GGT NS: FIB-4, hs-CRP, AST	(68)

F, female; M, male; MetS, metabolic syndrome; NAFLD, non-alcoholic fatty liver disease; TRE-x, eating within × hours, with the rest of the time being a fasting period; SOC, standard of care; LCD, low-carbohydrate diet; CR, caloric restriction; DCR, daily caloric restriction; LOV, Lacto-Ovo-vegetarian; ND, normal diet; N, nutrition recommendations; E, exercise recommendations; W, weight; WC, waist circumference; FM, fat mass; BFP, body fat percentage; LM, lean mass; VFA, visceral fat area; FPG, fasting blood glucose; FINS, fasting insulin; HbA1c, glycated hemoglobin; HOMA-IR, homeostatic model assessment of insulin resistance; IHTG, intrahepatic triglyceride; LDL-C, low-density lipoprotein cholesterol; HDL-C, high-density lipoprotein cholesterol; TC, total cholesterol; TG, triglycerides; SBP, systolic blood pressure; DBP, diastolic blood pressure; hs-CRP, high-sensitivity C-reactive protein; ALT, serum alanine aminotransferase; AST, aspartate aminotransferase; GGT, γ-Glutamyltransferase; CAP (FibroScan), Controlled Attenuation Parameter; LS (FibroScan), liver stiffness; FLI, fatty liver index; TNF-α, tumor necrosis factor alpha; UA, uric acid; PSQI, Pittsburgh sleep quality index; FIB-4, fibrosis-4 index.

Intervention group (vs. Control group): Comparison of the group's core intervention with its baseline or other interventions.

In clinical trials involving individuals with MetS, some studies indicate that despite spontaneous reductions in total caloric intake during TRE intervention, potential improvements in MetS-related markers are observed. However, further analysis suggests that these metabolic benefits show no significant correlation with caloric reduction or weight loss. For example, a small sample single-arm study of 19 MetS patients showed that TRE could still further reduce the relevant glycolipid indicators under the premise of combined drug use. Further analysis indicated these improvements were independent of weight changes. Specifically, the 12-week TRE-10 intervention significantly improved the patient's weight and WC, while reducing LDL-C, TC, and blood pressure levels, and the HbA1c of patients with elevated fasting blood glucose was also significantly reduced. The above results also provide a preliminary basis for the possible health benefits of TRE independent of weight loss, but given that this study is designed as a small sample single-arm study, this conclusion still needs to be verified by more high-quality research (61). Similarly, another 3-month RCT demonstrated that compared to the standard of care (SOC)-only group, the TRE combined with standard of care (SOC) nutrition counseling group not only significantly improved metabolic syndrome components, but also achieved superior weight management (average weight loss of 2.98 kg, with 75% fat loss and only 9% lean mass loss), and reduced calorie intake primarily from carbohydrates and fats, with no impact on protein intake. Notably, personalized TRE protocols shortened the eating window to 8–10 h (≥4 h shorter than baseline and ≥3 h before habitual sleep). Further analysis indicated that TRE's improvement in HbA1c among individuals with metabolic syndrome shows extremely weak correlations with calorie changes (*R*^2^ = 0.02) and weight loss (*R*^2^ = 0.07), further suggesting that calorie reduction is not the sole driving factor (62). In addition, trial results without measured caloric intake also demonstrated TRE's potential in reducing metabolic disease risk. Whether combined with a low-carb diet (LCD) or not, the 3-month TRE-8 intervention reduced the patients' body weight, HOMA-IR, TG, and uric acid (UA) indicators compared with the baseline. It is worth noting that the exploratory analysis of the TRE group showed that compared with lTRE-8, patients in the eTRE-8 (8:00–16:00) group could significantly reduce VFA. This suggests that the difference in the eating window period also causes differences, and e-TRE may be a more advantageous solution (63).

Among NAFLD populations, some trials have demonstrated that TRE improves body weight, body fat, inflammatory markers (such as hs-CRP and TNF-α), and liver-related assessments (including hepatic steatosis, liver fibrosis, and liver enzyme levels). Furthermore, long-term adherence to TRE-8 itself yields benefits by improving certain indicators, with these advantages potentially more closely linked to “calorie restriction.” For instance, a 12-month TRE-8 intervention reduced caloric intake compared to baseline while decreasing hepatic fat content (IHTG), improving liver fibrosis (LS, Liver Stiffness), liver enzyme levels, body weight, body fat, blood pressure, and indicators related to glycolipid metabolism. However, compared to DCR, TRE-8 did not yield particularly significant benefits without calorie reduction. Therefore, calorie control may be the primary mechanism at work when managing NAFLD through TRE protocols (64). The advantages of TRE over strict DCR are not significant, but studies suggest that TRE has benefits compared to combined interventions of SOC and exercise requirements (aerobic and resistance training). Specifically, one RCT demonstrated that compared to the SOC plus exercise group, TRE-8 significantly reduced hepatic steatosis (CAP, Controlled Attenuation Parameter), body weight, and waist circumference in NAFLD patients without reducing caloric intake. This suggests that altering meal timing or frequency alone may be a mechanism for improving visceral fat and hepatic steatosis (65). This finding aligns with previous research (66), concluding that “spreading meals across more frequent intervals while maintaining the same caloric intake increases hepatic fat.” Additionally, the combination of TRE-8 with specific dietary strategies demonstrated more pronounced effects. A 12-week isocaloric RCT was conducted to compare this combination (TRE-8 plus a low-sugar diet) with conventional dietary allocation. Results showed that the TRE-8 combined with a low-sugar diet intervention not only significantly improved hepatic steatosis score/CAP and liver enzyme levels (ALT, AST, and GGT) but also reduced anthropometric measurements, body composition parameters (W, WC, and FM), glycolipid-related indicators, and the inflammatory marker hs-CRP (67). Similarly, a 12-week TRE-8 combined with lacto-ovo vegetarian (LOV) intervention improved hepatic steatosis (FLI, Fatty Liver Index), body weight, waist circumference, glycolipid-related indicators (insulin levels, TG, and HDL-C), liver enzyme levels (ALT and GGT), and inflammatory status (TNF-α). However, no significant changes in liver fibrosis were observed (68).

In summary, standalone TRE interventions may yield limited effects in individuals with impaired metabolism. When combined with macronutrient guidance, disease-specific standard of care (SOC), or various dietary approaches (such as low-sugar diets or LOV), TRE may enhance efficacy through nutritional optimization, including sugar control and increased plant protein intake. Patients following eTRE-8 (8:00–16:00) may observe a reduction in visceral fat, or it may suggest that an earlier eating window combined with the TRE-8 protocol can achieve optimal effects.

### 4.4 Cancer

In an aging society, the threat of cancer is particularly prominent. With the increase in age, the decline in DNA repair ability, the weakening of immune surveillance, and the accumulation of epigenetic abnormalities make the risk of cancer in people over 60 is more than 10 times higher than that in young people. Preclinical studies have confirmed that TRE can delay the onset, progression, and metastasis of obesity-related breast, kidney, and lung cancers (69–71). The primary mechanisms likely involve restoring circadian rhythms of gene expression in tumors, synchronizing biological rhythms, or improving clock gene function. A large prospective cohort study of early-stage breast cancer patients showed that, after controlling for the confounding effect of “calorie restriction,” nighttime fasting duration < 13 h remained associated with a 36% increased risk of breast cancer recurrence. Moreover, longer nighttime fasting duration was closely linked to reduced HbA1c levels (72). However, the metabolic benefits of TRE for cancer survivors remain controversial. We summarized the intervention protocols of three single-arm TRE trials, and the effects of quality of life, fatigue, and psychological measures in cancer survivors (Table 4).

**Table 4:** Design, intervention protocol, and evaluation indicators for the time-restricted eating (TRE) trial in cancer participants.

**Type**	**Sample size and sex**	**Participants**	**Intervention group (protocol)**		**Control group (protocol)**		**Total calorie intake**	**Duration**	**Principal research outcomes**			**Reference**
			**Type of fasting**	**CR and NE**	**Comparison**	**CR and NE**			**Obesity indicators**	**Glycolipid indicators**	**Others**	
Single-Arm	39 36 F 3M	Cancer survivors	TRE-10 (self-selected eating window)	/	/	/	**TRE-10 (vs. baseline)** NS	2 weeks	**TRE-10 (vs baseline)** ↓ W	/	**TRE-10 (vs. baseline)** ↑ FACIT-F ↓ BFI	(73)
Single-Arm	22	Breast cancer survivors with risk factors for CVD mortality	TRE-8 (eat: 12:00–20:00) (working day)	/	/	/	**TRE-8 (vs. baseline)** ↓	8 weeks	**TRE-8 (vs. baseline)** ↓ W ↓ FM, ↓ VAT	**TRE-8 (vs. baseline)** NS: HDL-C, TC	**TRE-8 (vs. baseline)** NS: SBP ↓ Framingham CVD risk	(74)
Single-Arm	40 F	Breast cancer survivors	TRE-11 (POF: fasting durations of ≥13 h) (self-selected eating window)	/	/	/	/	12 weeks	**TRE-11 (vs. baseline)** ↓ BMI	**TRE-11 (vs. baseline)** NS: HbA1c, LDL-C, HDL-C, TG	**TRE-11 (vs. baseline)** ↑ FACIT-F ↓ HADS NS: FACT-G	(75)

F, female; M, male; TRE-x, eating within × hours, with the rest of the time being a fasting period; POF, prolonged overnight fasting; W, weight; FM, fat mass; VAT, visceral adipose tissue; HbA1c, glycated hemoglobin; LDL-C, low-density lipoprotein cholesterol; HDL-C, high-density lipoprotein cholesterol; TC, total cholesterol; TG, triglycerides; SBP, systolic blood pressure; FACIT-F, functional assessment of chronic illness therapy – fatigue; BFI, brief fatigue inventory; HADS, hospital anxiety and depression scale; FACT-G, functional assessment of cancer therapy – general.

Intervention group (vs. Control group): Comparison of the group's core intervention with its baseline or other interventions.

In clinical trials among cancer survivors, TRE emerges as a potential supportive therapeutic strategy. TRE not only reduces recurrence risk through its positive effects on body composition and cardiovascular risk factors but also mitigates acute and long-term side effects of cancer treatments, including cancer-related fatigue and quality of life aspects. For example, a 14-day TRE-10 dietary intervention yielded clinically meaningful improvements in body weight and fatigue symptoms (FACIT-F, Functional Assessment of Chronic Illness Therapy—Fatigue; BFI, Brief Fatigue Inventory) among cancer survivors without affecting caloric intake (73). Similarly, an 8-week TRE-8 (weekdays) study in obese/overweight breast cancer survivors who completed anthracycline chemotherapy 3 ± 1 years prior demonstrated spontaneous caloric intake reduction post-intervention, accompanied by concurrent decreases in body weight, total body fat mass, visceral adipose tissue, and 10-year Framingham cardiovascular disease risk. However, no significant changes were observed in the modifiable Framingham risk components (TC, HDL-C, and SBP), which indicates that there are individual differences in these indicators and the reduction in risk (74). In addition, a study of 40 breast cancer survivors showed that after a 12-week TRE-11 intervention program, the subjects' BMI, anxiety, and depression (HADS, Hospital Anxiety and Depression Scale) and fatigue symptoms (FACIT-F) were significantly improved (75). It should be noted that the role of TRE needs to be carefully evaluated in combination with the patient's nutritional status. In patients with advanced cancer malignancy, excessive restriction of the eating window may aggravate muscle breakdown, and it is necessary to weigh its dual impact on immune function improvement and nutritional support.

### 4.5 Others

At present, the multi-dimensional evaluation of healthy lifespan needs to take into account two aspects. It should not only pay attention to the core indicators related to the onset of chronic diseases and the risk of progression, such as weight management and glycolipid metabolism, but also include the assessment of cognitive, psychological, and other intrinsic abilities. In order to assess the health status of individuals in the aging process more comprehensively, we should not focus only on a single disease or a general quality of life (76). Preclinical studies showed that TRF may protect cognitive function and slow down cognitive decline by optimizing metabolic rhythms, reducing neuron loss, and the accumulation of Aβ plaques (77, 78). At the same time, some observational or interventional studies suggest that TRE may have a certain impact on the maintenance of memory function and the improvement of depressive symptoms. Its potential mechanisms are speculated to be related to insulin metabolism regulation, autophagy level regulation, neuroinflammatory response inhibition, and brain-derived neurotrophic factor (BDNF) expression regulation (79–81). These mechanisms may be involved in the regulation of neurogenesis and neuroplasticity.

In the cognitive field, some evidence shows that TRE has the potential to improve the decline of cognitive ability related to aging, but there are relatively few clinical studies in this regard. Specifically, a cross-sectional study analyzed the relationship between TRE and cognitive status in 883 Italian adults. The results showed that, even with no difference in calorie intake, individuals who followed the TRE-10 protocol were less likely to have cognitive impairment than those who did not practice TRE. This effect was particularly pronounced when food intake restriction aligned with circadian rhythms by starting earlier in the day (e-TRE or m-TRE) (82). Similarly, the 4-week eTRE-6 also improved the cognitive flexibility of college students without calorie reduction, which was manifested in the significant shortening of response times on the Trail Making Test-B (TMT-B). At the same time, this cognitive improvement was related to the ketone body in plasma (β-hydroxybutyrate) was negatively correlated, suggesting that ketones may play a role in it (83).

Current clinical evidence regarding the impact of TRE on human emotions and quality of life remains limited, but existing studies have shown a positive trend, and the effect of TRE may be independent of weight loss. Specifically, a 5-day isocaloric TRE-8 protocol improved participants' subjective happiness and optimized dietary patterns without weight loss. These benefits were not related to “calorie restriction” or weight loss (84). Meanwhile, although some studies did not report changes in calorie intake, they also reached similar conclusions. After the implementation of the 12-week TRE-8 protocol in the overweight population, the participants lost weight by approximately 3.7%. The SF-36 questionnaire revealed significant improvements in both the “emotional wellbeing” and “perceived health change” dimensions. Further analysis confirmed that improvement in quality of life was independent of weight loss (85). Another study similarly demonstrated that TRE's significant enhancement of health-related quality of life and sleep quality was not solely dependent on weight loss (86). In addition, a study conducted a 4-week TRE-8 intervention on 10 elderly people who were overweight, at risk of, or had mobility impairments (slow gait, < 1.0 m/s). The results showed participants experienced approximately 2.68% weight loss, along with clinically meaningful improvements in walking speed and quality of life. This suggests that TRE-8 is safe and feasible for this population, but the long-term effect still needs to be further verified (87).

### 4.6 Adherence and safety

Based on the results of a number of clinical studies, TRE showed high overall adherence in different populations, as well as good feasibility and safety, and no serious adverse reactions related to intervention were reported in all studies. Most clinical studies show that TRE is more adherent than other diet patterns (such as CR and LCD). The key is easy operation and high flexibility of the scheme. Specifically, compared with CR, the TRE protocol can retain the original dietary preferences of individuals without controlling calorie intake. At the same time, unlike LCD, ketogenic diets, and MD, TRE does not require forced changes to dietary structure, but only limits the eating time to reduce the difficulty of diet adjustment. In summary, participants avoid complex calorie counting, food selection, or dietary restrictions. They can also independently choose eating windows based on work schedules and sleep patterns, making it easier to integrate into daily routines and minimizing disruption to lifestyle habits. The TRE dietary pattern demonstrates significant adherence advantages in clinical interventions and may serve as an adjunct or alternative therapeutic approach for weight management and age-related NCDs such as T2D, MetS, NAFLD, cancer, and others.

Regarding safety, TRE has demonstrated good short-term safety. Only a small number of participants experienced minor and transient symptoms such as hunger, headaches, and fatigue during the initial phase of the intervention (particularly during the first few weeks of adaptation), and no intervention-related serious adverse events were reported in any study. However, attention should be paid to its long-term potential risks, such as possible lean body mass reduction and micronutrient deficiencies, and long-term adherence may decrease due to social factors.

## 5 Discussion

In the context of today's aging society, extending healthy lifespan is an important goal in the field of public health (88). However, the rapid development of modern society has brought severe challenges to metabolic health. Notably, the scientific definition of a healthy lifespan clearly states that individuals should remain free from major cognitive impairments, physical function limitations, and mental health problems after the age of 55. The irregular eating patterns throughout the day and the widespread use of artificial lighting at night not only lead to metabolic disorders (89) but also accelerate the progression of metabolic diseases and the aging process, seriously hindering the achievement of the goal of a healthy lifespan (90). There is no doubt about the impact of unhealthy lifestyles on health. As diet is a key modifiable factor for preventing non-communicable diseases (NCDs) and maintaining overall health during the aging process, the optimization of dietary intervention strategies is particularly crucial (91). As a dietary intervention mode of intermittent fasting, the core mode of TRE is in line with the concept of “chrononutrition” that has emerged in recent years (92). Significantly, this concept emphasizes that in addition to the quantity and quality of food, the timing of food intake is equally important for individual health.

The relationship between TRE and metabolic health has become a current research hotspot, but the inference of “TRE as a potential strategy for healthy lifespan” still needs to be verified in many aspects, such as high-quality and long-term follow-up research. We observe that in terms of weight management, existing studies show that TRE can show a certain downward trend in body weight, or can help individuals maintain a healthy body weight throughout life stages (93), which plays a crucial role in the process of achieving longevity and promoting a healthy lifespan (94). From the perspective of metabolic disorder regulation, TRE shows potential for preventing or correcting glycolipid metabolism disorders. Glycolipid-based metabolic disorders are the core pathological mechanisms for the development of age-related NCDs such as T2D, MetS, and cardiovascular and cerebrovascular diseases. Therefore, TRE may provide a potential intervention to reduce the incidence and associated risk of death by improving this metabolic disorder. At present, its regulatory effect may be to activate the multi-pathway collaborative regulation mechanism (95), thereby optimizing the overall metabolic state (96). In addition, TRE has also shown an improvement trend in terms of cognitive function, psychological state, and quality of life.

It is worth noting that the health benefits of TRE are jointly mediated by “calorie restriction” and “eating time restriction,” but there are certain differences in the health promotion mechanisms activated by the two. Among them, “calorie restriction” mainly activates fasting physiology and other mechanisms. The main contribution of the “eating time restriction” is the synchronization of peripheral and central biological clocks and other mechanisms. Additionally, it involves effects like reducing oxidative stress and improving gut microbiota composition. These effects may provide physiological foundations for maintaining homeostasis, intervening in the development of metabolic diseases, and delaying aging. Observational studies suggest that even after controlling for caloric intake, differences in fasting duration and eating window selection correlate with variations in weight loss, improvements in metabolic markers (e.g., HbA1c levels), or reduced breast cancer recurrence risk (97). However, this association does not establish direct causality. Some findings suggest that longer fasting periods and earlier eating windows may represent relatively advantageous TRE patterns. However, groups practicing longer fasts or earlier eating may be associated with higher Socioeconomic Status (SES; e.g., regular routines, higher education, strong health awareness, non-shift work), and higher SES itself is a strong predictor of longevity and health. Therefore, the observed association between TRE and health benefits in observational studies is likely driven predominantly by the powerful confounding factor of SES, rather than the effect of TRE itself.

After analyzing clinical study results from multiple TRE interventions, we found that its metabolic benefits depend on the combined regulation of both “calorie restriction” and “eating time restriction.” On one hand, extensive research confirms that TRE can spontaneously reduce an individual's calorie intake by approximately 20%, thereby achieving metabolic improvements through calorie restriction. The effect of this association with “calorie restriction” has been widely recognized. Simultaneously, some studies examining changes in calorie and nutrient intake before and after TRE interventions found that the spontaneous calorie reduction in TRE groups primarily stemmed from decreased carbohydrate (or total sugar) intake rather than protein. This trend suggests TRE may help optimize macronutrient patterns. On the other hand, even when controlling for “calorie restriction” (e.g., in studies with no difference in calorie intake or isocaloric conditions), some research still observed TRE's positive effects on relevant metabolic indicators. This suggests that TRE may offer metabolic benefits independent of calorie restriction, potentially through time-restricted eating mechanisms. It is worth noting that some studies suggest that TRE can bring metabolic benefits even independently of weight loss. For example, even without significant weight changes, metabolic benefits such as increased insulin sensitivity, reduced blood pressure, and decreased oxidative stress can be independently observed, suggesting deeper mechanisms beyond weight regulation. At the same time, the TRE of combined exercise has a more significant effect on blood lipid control. It is speculated that the reason may be the synergistic effect on optimizing the rhythm of lipid metabolism, promoting fat oxidation during fasting, and improving insulin sensitivity. Finally, we observed that in some studies, women experienced weight and waist circumference reduction but limited improvement in glycolipid metabolism markers. This is consistent with the notion that there are gender differences in energy metabolism, where females are more prone to fat accumulation and resistant to net fat loss, while males exhibit greater fat reduction and metabolic improvements under energy restriction (98). Thus, factors influencing TRE efficacy may relate to hormonal level differences between genders and variations in sensitivity to energy restriction across the hypothalamic–pituitary–gonadal axis (99).

In the TRE intervention protocols, the duration of fasting and the eating window are the core factors affecting intervention efficacy. Existing evidence suggests that the TRE-8 pattern may be the optimal choice. This may relate to the fact that excessively short fasting periods fail to activate beneficial mechanisms, while excessively long periods reduce compliance due to poor adaptability. In terms of the eating window time (100), the eTRE pattern has a more significant health improvement effect than the lTRE pattern (e.g., greater reduction in insulin resistance), which may be related to circadian rhythm. Overall, the existing evidence shows that the TRE-8 and the eTRE pattern may have more clinical advantages over other patterns.

At present, there are still many limitations in the clinical research on TRE, including flawed trial designs, insufficient evidence intensity, inadequate reporting and analysis of TRE intervention details, poor control of mixed factors, and incomplete representation of study populations. These limitations contribute to inconsistent findings and prevent the establishment of clear, unified conclusions. Most studies are short-term, small-sample clinical intervention studies, and lack exploration of the long-term effects and safety. At the same time, inadequate control of confounding factors, such as failure to strictly regulate calorie intake, meal frequency, and dietary quality (e.g., macronutrient composition and food variety), hinders precise assessment of the true effects of TRE. The existing TRE clinical research mainly focuses on overweight/obese people, and there are few studies on metabolically impaired people, such as MetS or T2D. This results in relatively healthy baseline metabolic status among most participants (bottom effects), thereby limiting the validity and generalizability of the TRE study findings. Moreover, in the few studies involving individuals with impaired metabolism, TRE intervention protocols were often combined with nutritional advice, other dietary patterns (e.g., DCR and LCD), or exercise recommendations, making it impossible to assess the effects of TRE alone. Because the current main research population has not fully covered other metabolic diseases (e.g., gout and hypothyroidism), the effectiveness of other metabolic-related results has not been fully verified. Furthermore, there is not enough attention paid to the gender differences in TRE metabolic benefits. Most studies lack a gender-stratification design and analysis, and some study subjects with impaired metabolism are mainly female, resulting in insufficient effectiveness of male data. This may be related to the higher prevalence of certain diseases (e.g., hormone-related conditions like female-specific obesity and postmenopausal metabolic syndrome) among women.

In view of the current limitations, future TRE clinical intervention research should optimize the experimental design from many aspects. This includes focusing on isocaloric designs to distinguish the respective contributions of “calorie restriction” and “eating time restriction” within TRE effects, or conducting long-term, large-sample studies to explore TRE's long-term efficacy and safety. Rhythm-related assessments and indicators can be added to the research program in the future. For example, introduce additional time-rhythm related evaluations at the baseline states, identifying participants' chronotypes (morning larks/night owls) and sleep-wake cycles through sleep logs, wearable devices, or other tools. Regarding intervention protocols, on one hand, potential confounding factors like calorie intake, meal frequency, and dietary quality should be strictly controlled to minimize their impact on study outcomes. On the other hand, formulate TRE protocols (fasting duration and eating window duration) according to the baseline state of the population to improve the effectiveness of the intervention. In addition, attention should be paid to the inclusion of gender-balanced groups in the design stage to avoid gender differences affecting the research results.

In summary, although the role of TRE on healthy aging needs to be further explored, its ability to reduce chronic inflammation and oxidative stress, improve intestinal barrier dysfunction, and maintain metabolic homeostasis (101) may be a key factor in delaying the physiological and pathological process of aging. At present, there are many studies on other diet patterns in evaluating the effect of improving aging-related diseases and delaying the aging process, such as CR, MD, and anti-inflammatory diets (102). However, we note that current TRE research predominantly focuses on specific disease domains, with outcomes primarily centered on body weight and related metabolic indicators. There is a near absence of studies investigating long-term human health outcomes (e.g., incidence of adverse cardiovascular and cerebrovascular events). While we observe TRE's potential in improving obesity, metabolic health, and related risk factors alongside quality of life, its true impact on long-term societal health outcomes and healthy longevity remains an unresolved question. Future research could integrate multidimensional concepts of healthy lifespan (e.g., cognitive and emotional aspects) and multiple dimensions of quality of life (103) to assess the association between TRE dietary patterns and healthy lifespan.

## 6 Conclusion

The TRE dietary intervention pattern holds significant clinical and public health implications regarding its efficacy and safety in promoting a healthy lifespan. Existing clinical studies suggest that TRE may help maintain a healthy weight, improve glucose and lipid metabolism disorders, and thereby reduce the risk of certain chronic diseases, as well as exert positive effects on quality of life. However, the long-term safety of TRE still requires further evaluation. Furthermore, constrained by the design and evidence strength of current studies, future high-quality research is needed to validate these findings.

## References

[1] GBD 2017 Causes of Death Collaborators. Global, regional, and national age-sex-specific mortality for 282 causes of death in 195 countries and territories, 1980-2017: a systematic analysis for the Global Burden of Disease Study 2017. Lancet. (2018) 392:1736–88. doi: 10.1016/S0140-6736(18)32203-7 30496103 PMC6227606

[2] Jivraj S Goodman A Pongiglione B Ploubidis GB. Living longer but not necessarily healthier: the joint progress of health and mortality in the working-age population of England. Popul Stud. (2020) 74:399–414. doi: 10.1080/00324728.2020.1767297 32659174

[3] Beard JR Officer A de Carvalho IA Sadana R Pot AM Michel JP . The World report on ageing and health: a policy framework for healthy ageing. Lancet. (2016) 387:2145–54. doi: 10.1016/S0140-6736(15)00516-4 26520231 PMC4848186

[4] Green CL Lamming DW Fontana L. Molecular mechanisms of dietary restriction promoting health and longevity. Nat Rev Mol Cell Biol. (2022) 23:56–73. doi: 10.1038/s41580-021-00411-4 34518687 PMC8692439

[5] Xiao YL Gong Y Qi YJ Shao ZM Jiang YZ. Effects of dietary intervention on human diseases: molecular mechanisms and therapeutic potential. Signal Transduct Target Ther. (2024) 9:59. doi: 10.1038/s41392-024-01771-x 38462638 PMC10925609

[6] Vasim I Majeed CN DeBoer MD. Intermittent fasting and metabolic health. Nutrients. (2022) 14:631. doi: 10.3390/nu14030631 35276989 PMC8839325

[7] Rynders CA Thomas EA Zaman A Pan Z Catenacci VA Melanson EL. Effectiveness of intermittent fasting and time-restricted feeding compared to continuous energy restriction for weight loss. Nutrients. (2019) 11:2442. doi: 10.3390/nu11102442 31614992 PMC6836017

[8] Petersen MC Gallop MR Flores Ramos S Zarrinpar A Broussard JL Chondronikola M . Complex physiology and clinical implications of time-restricted eating. Physiol Rev. (2022) 102:1991–2034. doi: 10.1152/physrev.00006.2022 35834774 PMC9423781

[9] Challet E. The circadian regulation of food intake. Nat Revi Endocrinol. (2019) 15:393–405. doi: 10.1038/s41574-019-0210-x 31073218

[10] Zeb F Wu X Chen L Fatima S Haq IU Chen A . Effect of time-restricted feeding on metabolic risk and circadian rhythm associated with gut microbiome in healthy males. Br J Nutr. (2020) 123:1216–26. doi: 10.1017/S0007114519003428 31902372

[11] Bray MS Ratcliffe WF Grenett MH Brewer RA Gamble KL Young ME. Quantitative analysis of light-phase restricted feeding reveals metabolic dyssynchrony in mice. Int J Obes. (2013) 37:843–52. doi: 10.1038/ijo.2012.137 22907695 PMC3505273

[12] Yasumoto Y Hashimoto C Nakao R Yamazaki H Hiroyama H Nemoto T . Short-term feeding at the wrong time is sufficient to desynchronize peripheral clocks and induce obesity with hyperphagia, physical inactivity and metabolic disorders in mice. Metabolism. (2016) 65:714–27. doi: 10.1016/j.metabol.2016.02.003 27085778

[13] Mattson MP Moehl K Ghena N Schmaedick M Cheng A. Intermittent metabolic switching, neuroplasticity and brain health. Nat Rev Neurosci. (2018) 19:63–80. doi: 10.1038/nrn.2017.156 29321682 PMC5913738

[14] Adamovich Y Rousso-Noori L Zwighaft Z Neufeld-Cohen A Golik M Kraut-Cohen J . Circadian clocks and feeding time regulate the oscillations and levels of hepatic triglycerides. Cell Metab. (2014) 19:319–30. doi: 10.1016/j.cmet.2013.12.016 24506873 PMC4261230

[15] Bugge A Feng D Everett LJ Briggs ER Mullican SE Wang F . Rev-erbα and Rev-erbβ coordinately protect the circadian clock and normal metabolic function. Genes Dev. (2012) 26:657–67. doi: 10.1101/gad.186858.112 22474260 PMC3323877

[16] Raefsky SM Mattson MP. Adaptive responses of neuronal mitochondria to bioenergetic challenges: roles in neuroplasticity and disease resistance. Free Radic Biol Med. (2017) 102:203–16. doi: 10.1016/j.freeradbiomed.2016.11.045 27908782 PMC5209274

[17] Mattson MP. The cyclic metabolic switching theory of intermittent fasting. Nat Metab. (2025) 7:665–78. doi: 10.1038/s42255-025-01254-5 40087409

[18] Jomova K Alomar SY Alwasel SH Nepovimova E Kuca K Valko M. Several lines of antioxidant defense against oxidative stress: antioxidant enzymes, nanomaterials with multiple enzyme-mimicking activities, and low-molecular-weight antioxidants. Arch Toxicol. (2024) 98:1323–67. doi: 10.1007/s00204-024-03696-4 38483584 PMC11303474

[19] Ella K Sudy ÁR Búr Z Koós B Kisiczki ÁS Mócsai A . Time restricted feeding modifies leukocyte responsiveness and improves inflammation outcome. Front Immunol. (2022) 13:924541. doi: 10.3389/fimmu.2022.924541 36405720 PMC9666763

[20] Malinowski B Zalewska K Wesierska A Sokołowska MM Socha M Liczner G . Intermittent fasting in cardiovascular disorders-an overview. Nutrients. (2019) 11:673. doi: 10.3390/nu11030673 30897855 PMC6471315

[21] Kaczmarek JL Musaad SM Holscher HD. Time of day and eating behaviors are associated with the composition and function of the human gastrointestinal microbiota. Am J Clin Nutr. (2017) 106:1220–31. doi: 10.3945/ajcn.117.156380 28971851

[22] Li L Su Y Li F Wang Y Ma Z Li Z . The effects of daily fasting hours on shaping gut microbiota in mice. BMC Microbiol. (2020) 20:65. doi: 10.1186/s12866-020-01754-2 32209070 PMC7092480

[23] van der Merwe M Sharma S Caldwell JL Smith NJ Gomes CK Bloomer RJ . Time of feeding alters obesity-associated parameters and gut bacterial communities, but not fungal populations, in C57BL/6 male mice. Curr Dev Nutr. (2020) 4:nzz145. doi: 10.1093/cdn/nzz145 32025616 PMC6992463

[24] Di Vincenzo F Del Gaudio A Petito V Lopetuso LR Scaldaferri F. Gut microbiota, intestinal permeability, and systemic inflammation: a narrative review. Intern Emerg Med. (2024) 19:275–93. doi: 10.1007/s11739-023-03374-w 37505311 PMC10954893

[25] Lim MY Nam YD. Gut microbiome in healthy aging versus those associated with frailty. Gut Microbes. (2023) 15:2278225. doi: 10.1080/19490976.2023.2278225 37968837 PMC10730223

[26] Dogra S Dunstan DW Sugiyama T Stathi A Gardiner PA Owen N. Active aging and public health: evidence, implications, and opportunities. Annu Rev Public Health. (2022) 43:439–59. doi: 10.1146/annurev-publhealth-052620-091107 34910580

[27] Hatori M Vollmers C Zarrinpar A DiTacchio L Bushong EA Gill S . Time-restricted feeding without reducing caloric intake prevents metabolic diseases in mice fed a high-fat diet. Cell Metab. (2012) 15:848–60. doi: 10.1016/j.cmet.2012.04.019 22608008 PMC3491655

[28] Brandhorst S Choi IY Wei M Cheng CW Sedrakyan S Navarrete G . A periodic diet that mimics fasting promotes multi-system regeneration, enhanced cognitive performance, and healthspan. Cell Metab. (2015) 22:86–99. doi: 10.1016/j.cmet.2015.05.012 26094889 PMC4509734

[29] Cienfuegos S Gabel K Kalam F Ezpeleta M Wiseman E Pavlou V . Effects of 4- and 6-h time-restricted feeding on weight and cardiometabolic health: a randomized controlled trial in adults with obesity. Cell Metab. (2020) 32:366–78.e3. doi: 10.1016/j.cmet.2020.06.018 32673591 PMC9407646

[30] Liu D Huang Y Huang C Yang S Wei X Zhang P . Calorie restriction with or without time-restricted eating in weight loss. N Engl J Med. (2022) 386:1495–504. doi: 10.1056/NEJMoa2114833 35443107

[31] Lin S Cienfuegos S Ezpeleta M Gabel K Pavlou V Mulas A . Time-restricted eating without calorie counting for weight loss in a racially diverse population: a randomized controlled trial. Ann Intern Med. (2023) 176:885–95. doi: 10.7326/M23-0052 37364268 PMC11192144

[32] Lowe DA Wu N Rohdin-Bibby L Moore AH Kelly N Liu YE . Effects of time-restricted eating on weight loss and other metabolic parameters in women and men with overweight and obesity: the TREAT Randomized Clinical Trial. JAMA Intern Med. (2020) 180:1491–9. doi: 10.1001/jamainternmed.2020.4153 32986097 PMC7522780

[33] Herz D Karl S Weiß J Zimmermann P Haupt S Zimmer RT . Effects of different types of intermittent fasting interventions on metabolic health in healthy individuals (EDIF): a randomised trial with a controlled-run in phase. Nutrients. (2024) 16:1114. doi: 10.3390/nu16081114 38674802 PMC11054438

[34] Xie Z Sun Y Ye Y Hu D Zhang H He Z . Randomized controlled trial for time-restricted eating in healthy volunteers without obesity. Nat Commun. (2022) 13:1003. doi: 10.1038/s41467-022-28662-5 35194047 PMC8864028

[35] Sampieri A Paoli A Spinello G Santinello E Moro T. Impact of daily fasting duration on body composition and cardiometabolic risk factors during a time-restricted eating protocol: a randomized controlled trial. J Transl Med. (2024) 22:1086. doi: 10.1186/s12967-024-05849-6 39614235 PMC11607941

[36] Chen Y Li X Yang M Jia C He Z Zhou S . Time-restricted eating reveals a “younger” immune system and reshapes the intestinal microbiome in human. Redox Biol. (2024) 78:103422. doi: 10.1016/j.redox.2024.103422 39561680 PMC11616606

[37] Liu Z Dai X Zhang H Shi R Hui Y Jin X . Gut microbiota mediates intermittent-fasting alleviation of diabetes-induced cognitive impairment. Nat Commun. (2020) 11:855. doi: 10.1038/s41467-020-14676-4 32071312 PMC7029019

[38] Peeke PM Greenway FL Billes SK Zhang D Fujioka K. Effect of time restricted eating on body weight and fasting glucose in participants with obesity: results of a randomized, controlled, virtual clinical trial. Nutr Diabetes. (2021) 11:6. doi: 10.1038/s41387-021-00149-0 33446635 PMC7809455

[39] McAllister MJ Pigg BL Renteria LI Waldman HS. Time-restricted feeding improves markers of cardiometabolic health in physically active college-age men: a 4-week randomized pre-post pilot study. Nutr Res. (2020) 75:32–43. doi: 10.1016/j.nutres.2019.12.001 31955013

[40] Lin YJ Wang YT Chan LC Chu NF. Effect of time-restricted feeding on body composition and cardio-metabolic risk in middle-aged women in Taiwan. Nutrition. (2022) 93:111504. doi: 10.1016/j.nut.2021.111504 34763309

[41] Schroder JD Falqueto H Mânica A Zanini D de Oliveira T de Sá CA . Effects of time-restricted feeding in weight loss, metabolic syndrome and cardiovascular risk in obese women. J Transl Med. (2021) 19:3. doi: 10.1186/s12967-020-02687-0 33407612 PMC7786967

[42] Tan LJ Shin S. Impact of eating duration on weight management, sleeping quality, and psychological stress: a pilot study. J Nutr Biochem. (2025) 137:109835. doi: 10.1016/j.jnutbio.2024.109835 39701471

[43] Domaszewski P Konieczny M Dybek T Łukaniszyn-Domaszewska K Anton S Sadowska-Krepa E . Comparison of the effects of six-week time-restricted eating on weight loss, body composition, and visceral fat in overweight older men and women. Exp Gerontol. (2023) 174:112116. doi: 10.1016/j.exger.2023.112116 36739795

[44] Moro T Tinsley G Pacelli FQ Marcolin G Bianco A Paoli A. Twelve months of time-restricted eating and resistance training improves inflammatory markers and cardiometabolic risk factors. Med Sci Sports Exerc. (2021) 53:2577–85. doi: 10.1249/MSS.0000000000002738 34649266 PMC10115489

[45] Haganes KL Silva CP Eyjólfsdóttir SK Steen S Grindberg M Lydersen S . Time-restricted eating and exercise training improve HbA1c and body composition in women with overweight/obesity: a randomized controlled trial. Cell Metab. (2022) 34:1457–71.e4. doi: 10.1016/j.cmet.2022.09.003 36198292

[46] Mekary RA Giovannucci E Willett WC van Dam RM Hu FB. Eating patterns and type 2 diabetes risk in men: breakfast omission, eating frequency, and snacking. Am J Clin Nutr. (2012) 95:1182–9. doi: 10.3945/ajcn.111.028209 22456660 PMC3325839

[47] Asher G Sassone-Corsi P. Time for food: the intimate interplay between nutrition, metabolism, and the circadian clock. Cell. (2015) 161:84–92. doi: 10.1016/j.cell.2015.03.015 25815987

[48] Parr EB Radford BE Hall RC Steventon-Lorenzen N Flint SA Siviour Z . Comparing the effects of time-restricted eating on glycaemic control in people with type 2 diabetes with standard dietetic practice: a randomised controlled trial. Diabetes Res Clin Pract. (2024) 217:111893. doi: 10.1016/j.diabres.2024.111893 39414086

[49] Pavlou V Cienfuegos S Lin S Ezpeleta M Ready K Corapi S . Effect of time-restricted eating on weight loss in adults with type 2 diabetes: a randomized clinical trial. JAMA Netw. Open. (2023) 6:e2339337. doi: 10.1001/jamanetworkopen.2023.39337 37889487 PMC10611992

[50] Pavlou V Lin S Cienfuegos S Ezpeleta M Runchey MC Corapi S . Effect of time-restricted eating on sleep in type 2 diabetes. Nutrients. (2024) 16:2742. doi: 10.3390/nu16162742 39203878 PMC11356804

[51] Che T Yan C Tian D Zhang X Liu X Wu Z. Time-restricted feeding improves blood glucose and insulin sensitivity in overweight patients with type 2 diabetes: a randomised controlled trial. Nutr Metab. (2021) 18:88. doi: 10.1186/s12986-021-00613-9 34620199 PMC8499480

[52] Quist JS Pedersen HE Jensen MM Clemmensen KKB Bjerre N Ekblond TS . Effects of 3 months of 10-h per-day time-restricted eating and 3 months of follow-up on bodyweight and cardiometabolic health in Danish individuals at high risk of type 2 diabetes: the RESET single-centre, parallel, superiority, open-label, randomised controlled trial. Lancet Healthy Longev. (2024) 5:e314–25. doi: 10.1016/S2666-7568(24)00028-X 38588687

[53] Suthutvoravut U Anothaisintawee T Boonmanunt S Pramyothin S Siriyothin S Attia J . Efficacy of time-restricted eating and behavioral economic intervention in reducing fasting plasma glucose, HbA1c, and cardiometabolic risk factors in patients with impaired fasting glucose: a randomized controlled trial. Nutrients. (2023) 15:4233. doi: 10.3390/nu15194233 37836517 PMC10574576

[54] Hegedus E Vu MH Salvy SJ Bakhsh J Goran MI Raymond JK . Randomized controlled feasibility trial of late 8-hour time-restricted eating for adolescents with type 2 diabetes. J Acad Nutr Diet. (2024) 124:1014–28. doi: 10.1016/j.jand.2023.10.012 39464252 PMC11507361

[55] Sutton EF Beyl R Early KS Cefalu WT Ravussin E Peterson CM. Early time-restricted feeding improves insulin sensitivity, blood pressure, and oxidative stress even without weight loss in men with prediabetes. Cell Metab. (2018) 27:1212–21.e3. doi: 10.1016/j.cmet.2018.04.010 29754952 PMC5990470

[56] Del Ben M Polimeni L Brancorsini M Di Costanzo A D'Erasmo L Baratta F . Non-alcoholic fatty liver disease, metabolic syndrome and patatin-like phospholipase domain-containing protein3 gene variants. Eur J Intern Med. (2014) 25:566–70. doi: 10.1016/j.ejim.2014.05.012 24947770

[57] Yki-Järvinen H. Non-alcoholic fatty liver disease as a cause and a consequence of metabolic syndrome. Lancet Diabetes Endocrinol. (2014) 2:901–10. doi: 10.1016/S2213-8587(14)70032-4 24731669

[58] Bence KK Birnbaum MJ. Metabolic drivers of non-alcoholic fatty liver disease. Mol Metab. (2021) 50:101143. doi: 10.1016/j.molmet.2020.101143 33346069 PMC8324696

[59] Gong Y Zhang H Feng J Ying L Ji M Wei S . Time-restricted feeding improves metabolic syndrome by activating thermogenesis in brown adipose tissue and reducing inflammatory markers. Front Immunol. (2025) 16:1501850. doi: 10.3389/fimmu.2025.1501850 39925816 PMC11802511

[60] Wilson RB Zhang R Chen YJ Peters KM Sawyez CG Sutherland BG . Two-week isocaloric time-restricted feeding decreases liver inflammation without significant weight loss in obese mice with non-alcoholic fatty liver disease. Int J Mol Sci. (2020) 21:9156. doi: 10.3390/ijms21239156 33271781 PMC7730100

[61] Wilkinson MJ Manoogian ENC Zadourian A Lo H Fakhouri S Shoghi A . Ten-hour time-restricted eating reduces weight, blood pressure, and atherogenic lipids in patients with metabolic syndrome. Cell Metab. (2020) 31:92–104.e5. doi: 10.1016/j.cmet.2019.11.004 31813824 PMC6953486

[62] Manoogian ENC Wilkinson MJ O'Neal M Laing K Nguyen J Van D . Time-restricted eating in adults with metabolic syndrome: a randomized controlled trial. Ann Intern Med. (2024) 177:1462–70. doi: 10.7326/M24-0859 39348690 PMC11929607

[63] He M Wang J Liang Q Li M Guo H Wang Y . Time-restricted eating with or without low-carbohydrate diet reduces visceral fat and improves metabolic syndrome: a randomized trial. Cell Rep Med. (2022) 3:100777. doi: 10.1016/j.xcrm.2022.100777 36220069 PMC9589024

[64] Wei X Lin B Huang Y Yang S Huang C Shi L . Effects of time-restricted eating on nonalcoholic fatty liver disease: the TREATY-FLD randomized clinical trial. JAMA Netw Open. (2023) 6:e233513. doi: 10.1001/jamanetworkopen.2023.3513 36930148 PMC10024204

[65] Feehan J Mack A Tuck C Tchongue J Holt DQ Sievert W . Time-restricted fasting improves liver steatosis in non-alcoholic fatty liver disease-a single blinded crossover trial. Nutrients. (2023) 15:4870. doi: 10.3390/nu15234870 38068729 PMC10708421

[66] Koopman KE Caan MW Nederveen AJ Pels A Ackermans MT Fliers E . Hypercaloric diets with increased meal frequency, but not meal size, increase intrahepatic triglycerides: a randomized controlled trial. Hepatology. (2014) 60:545–53. doi: 10.1002/hep.27149 24668862 PMC4265261

[67] Kord-Varkaneh H Salehi-Sahlabadi A Tinsley GM Santos HO Hekmatdoost A. Effects of time-restricted feeding (16/8) combined with a low-sugar diet on the management of non-alcoholic fatty liver disease: a randomized controlled trial. Nutrition. (2023) 105:111847. doi: 10.1016/j.nut.2022.111847 36257081

[68] Shafiee M Sadeghi A Ghafouri-Taleghani F Nilghaz M Ghods M Narimani B . Effects of time restricted feeding combined with Lacto Ovo vegetarian diet on metabolic associated fatty liver disease management: a randomized clinical trial. Sci Rep. (2025) 15:4463. doi: 10.1038/s41598-025-88773-z 39915600 PMC11803106

[69] Das M Ellies LG Kumar D Sauceda C Oberg A Gross E . Time-restricted feeding normalizes hyperinsulinemia to inhibit breast cancer in obese postmenopausal mouse models. Nat Commun. (2021) 12:565. doi: 10.1038/s41467-020-20743-7 33495474 PMC7835248

[70] Turbitt WJ Orlandella RM Gibson JT Peterson CM Norian LA. Therapeutic time-restricted feeding reduces renal tumor bioluminescence in mice but fails to improve anti-CTLA-4 efficacy. Anticancer Res. (2020) 40:5445–56. doi: 10.21873/anticanres.14555 32988866 PMC7957951

[71] Shi D Fang G Chen Q Li J Ruan X Lian X. Six-hour time-restricted feeding inhibits lung cancer progression and reshapes circadian metabolism. BMC Med. (2023) 21:417. doi: 10.1186/s12916-023-03131-y 37924048 PMC10625271

[72] Marinac CR Nelson SH Breen CI Hartman SJ Natarajan L Pierce JP . Prolonged nightly fasting and breast cancer prognosis. JAMA Oncol. (2016) 2:1049–55. doi: 10.1001/jamaoncol.2016.0164 27032109 PMC4982776

[73] Kleckner AS Altman BJ Reschke JE Kleckner IR Culakova E Dunne RF . Time-restricted eating to address cancer-related fatigue among cancer survivors: a single-arm pilot study. J Integr Oncol. (2022) 11:379. doi: 10.21203/rs.3.rs-1433008/v1 36131848 PMC9489052

[74] Kirkham AA Ford KL Topolnyski J Da Silva BR Paterson DI Prado CM . Time-restricted eating to reduce cardiovascular risk among older breast cancer survivors: a single-arm feasibility study. JACC Cardio Oncol. (2022) 4:276–78. doi: 10.1016/j.jaccao.2022.03.002 35818550 PMC9270634

[75] O'Donnell E Shapiro Y Comander A Isakoff S Moy B Spring L . Pilot study to assess prolonged overnight fasting in breast cancer survivors (longfast). Breast Cancer Res Treat. (2022) 193:579–87. doi: 10.1007/s10549-022-06594-4 35441995

[76] Cesari M Carvalho IA Thiyagarajan JA Cooper C Martin FC Reginster JY . Evidence for the domains supporting the construct of intrinsic capacity. J Gerontol A Biol Sci Med Sci. (2018) 73:1653–60. doi: 10.1093/gerona/gly011 29408961

[77] Halagappa VK Guo Z Pearson M Matsuoka Y Cutler RG Laferla FM . Intermittent fasting and caloric restriction ameliorate age-related behavioral deficits in the triple-transgenic mouse model of Alzheimer's disease. Neurobiol Dis. (2007) 26:212–20. doi: 10.1016/j.nbd.2006.12.019 17306982

[78] Bruce-Keller AJ Umberger G McFall R Mattson MP. Food restriction reduces brain damage and improves behavioral outcome following excitotoxic and metabolic insults. Ann Neurol. (1999) 45:8–15. doi: 10.1002/1531-8249(199901)45:1<8::AID-ART4>3.0.CO;2-V 9894871

[79] Currenti W Godos J Castellano S Mogavero MP Ferri R Caraci F . Time restricted feeding and mental health: a review of possible mechanisms on affective and cognitive disorders. Int J Food Sci Nutr. (2021) 72:723–33. doi: 10.1080/09637486.2020.1866504 33356688

[80] Igwe O Sone M Matveychuk D Baker GB Dursun SM. A review of effects of calorie restriction and fasting with potential relevance to depression. Prog Neuropsychopharmacol Biol Psychiatry. (2021) 111:110206. doi: 10.1016/j.pnpbp.2020.110206 33316333

[81] Seidler K Barrow M. Intermittent fasting and cognitive performance - Targeting BDNF as potential strategy to optimise brain health. Front Neuroendocrinol. (2022) 65:100971. doi: 10.1016/j.yfrne.2021.100971 34929259

[82] Currenti W Godos J Castellano S Caruso G Ferri R Caraci F . Association between time restricted feeding and cognitive status in older Italian adults. Nutrients. (2021) 13:191. doi: 10.3390/nu13010191 33435416 PMC7827225

[83] Mayra ST Kravat N Chondropoulos K De Leon A Johnston CS. Early time-restricted eating may favorably impact cognitive acuity in university students: a randomized pilot study. Nutr Res. (2022) 108:1–8. doi: 10.1016/j.nutres.2022.10.002 36351326

[84] Parr EB Devlin BL Radford BE Hawley JA. A Delayed morning and earlier evening time-restricted feeding protocol for improving glycemic control and dietary adherence in men with overweight/obesity: a randomized controlled trial. Nutrients. (2020) 12:505. doi: 10.3390/nu12020505 32079327 PMC7071240

[85] Crose A Alvear A Singroy S Wang Q Manoogian E Panda S . Time-restricted eating improves quality of life measures in overweight humans. Nutrients. (2021) 13:1430. doi: 10.3390/nu13051430 33922683 PMC8146708

[86] Kesztyüs D Fuchs M Cermak P Kesztyüs T. Associations of time-restricted eating with health-related quality of life and sleep in adults: a secondary analysis of two pre-post pilot studies. BMC Nutr. (2020) 6:76. doi: 10.1186/s40795-020-00402-2 33327959 PMC7745395

[87] Anton SD Lee SA Donahoo WT McLaren C Manini T Leeuwenburgh C . The effects of time restricted feeding on overweight, older adults: a pilot study. Nutrients. (2019) 11:1500. doi: 10.3390/nu11071500 31262054 PMC6682944

[88] Zhang K Ma Y Luo Y Song Y Xiong G Ma Y . Metabolic diseases and healthy aging: identifying environmental and behavioral risk factors and promoting public health. Front Public Health. (2023) 11:1253506. doi: 10.3389/fpubh.2023.1253506 37900047 PMC10603303

[89] Muscogiuri G Poggiogalle E Barrea L Tarsitano MG Garifalos F Liccardi A . Exposure to artificial light at night: a common link for obesity and cancer? Eur J Cancer. (2022) 173:263–75. doi: 10.1016/j.ejca.2022.06.007 35940056

[90] Fan M Yuan J Zhang S Fu Q Lu D Wang Q . Association between outdoor artificial light at night and metabolic diseases in middle-aged to older adults-the CHARLS survey. Front Public Health. (2025) 13:1515597. doi: 10.3389/fpubh.2025.1515597 40115347 PMC11922846

[91] Duan H Pan J Guo M Li J Yu L Fan L. Dietary strategies with anti-aging potential: dietary patterns and supplements. Food Res Int. (2022) 158:111501. doi: 10.1016/j.foodres.2022.111501 35840210

[92] Adafer R Messaadi W Meddahi M Patey A Haderbache A Bayen S . Food timing, circadian rhythm and chrononutrition: a systematic review of time-restricted eating's effects on human health. Nutrients. (2020) 12:3770. doi: 10.3390/nu12123770 33302500 PMC7763532

[93] Zheng Y Manson JE Yuan C Liang MH Grodstein F Stampfer MJ . Associations of weight gain from early to middle adulthood with major health outcomes later in life. JAMA. (2017) 318:255–69. doi: 10.1001/jama.2017.7092 28719691 PMC5817436

[94] Veronese N Li Y Manson JE Willett WC Fontana L Hu FB. Combined associations of body weight and lifestyle factors with all cause and cause specific mortality in men and women: prospective cohort study. BMJ. (2016) 355:i5855. doi: 10.1136/bmj.i5855 27884868 PMC5122318

[95] de Oliveira Melo NC Cuevas-Sierra A Souto VF Martínez JA. Biological rhythms, chrono-nutrition, and gut microbiota: epigenomics insights for precision nutrition and metabolic health. Biomolecules. (2024) 14:559. doi: 10.3390/biom14050559 38785965 PMC11117887

[96] Zhang M Zhou C Li X Li H Han Q Chen Z . Interactions between gut microbiota, host circadian rhythms, and metabolic diseases. Adv Nutr. (2025) 16:100416. doi: 10.1016/j.advnut.2025.100416 40139315 PMC12148639

[97] Garaulet M Gómez-Abellán P Alburquerque-Béjar JJ Lee YC Ordovás JM Scheer FA. Timing of food intake predicts weight loss effectiveness. Int J Obes. (2013) 37:604–11. doi: 10.1038/ijo.2012.229 23357955 PMC3756673

[98] Chaix A Deota S Bhardwaj R Lin T Panda S. Sex- and age-dependent outcomes of 9-hour time-restricted feeding of a Western high-fat high-sucrose diet in C57BL/6J mice. Cell Rep. (2021) 36:109543. doi: 10.1016/j.celrep.2021.109543 34407415 PMC8500107

[99] Mauvais-Jarvis F. Sex differences in energy metabolism: natural selection, mechanisms and consequences. Nat Rev Nephrol. (2024) 20:56–69. doi: 10.1038/s41581-023-00781-2 37923858

[100] Liu J Yi P Liu F. The effect of early time-restricted eating vs later time-restricted eating on weight loss and metabolic health. J Clin Endocrinol Metab. (2023) 108:1824–34. doi: 10.1210/clinem/dgad036 36702768

[101] Rera M Clark RI Walker DW. Intestinal barrier dysfunction links metabolic and inflammatory markers of aging to death in Drosophila. Proc Natl Acad Sci USA. (2012) 109:21528–33. doi: 10.1073/pnas.1215849110 23236133 PMC3535647

[102] Yeung SSY Kwan M Woo J. Healthy diet for healthy aging. Nutrients. (2021) 13:4310. doi: 10.3390/nu13124310 34959862 PMC8707325

[103] Hu FB. Diet strategies for promoting healthy aging and longevity: an epidemiological perspective. J Intern Med. (2024) 295:508–31. doi: 10.1111/joim.13728 37867396 PMC10939982

